# Online functional connectivity analysis of large all-to-all networks in MNE Scan

**DOI:** 10.1162/imag_a_00296

**Published:** 2024-09-25

**Authors:** Lorenz Esch, Jinlong Dong, Matti Hämäläinen, Daniel Baumgarten, Jens Haueisen, Johannes Vorwerk

**Affiliations:** Institute of Biomedical Engineering and Informatics, Technische Universität Ilmenau, Ilmenau, Germany; Athinoula A. Martinos Center for Biomedical Imaging, Massachusetts General Hospital, Harvard Medical School, Boston, MA, United States; Department of Neuroscience and Biomedical Engineering, Aalto University School of Schience, Espoo, Finland; Department of Radiology, Massachusetts General Hospital, Harvard Medical School, Boston, MA, United States; Institute of Electrical and Biomedical Engineering, UMIT TIROL—Private University for Health Sciences and Health Technology, Hall in Tirol, Austria; Institute of Mechatronics, University of Innsbruck, Innsbruck, Austria

**Keywords:** electroencephalography, magnetoencephalography, connectivity analysis, source localization, and online processing

## Abstract

The analysis of electroencephalography (EEG)/magnetoencephalography (MEG) functional connectivity has become an important tool in neuroscience. Especially the high time resolution of EEG/MEG enables important insight into the functioning of the human brain. To date, functional connectivity is commonly estimated offline, that is, after the conclusion of the experiment. However, online computation of functional connectivity has the potential to enable unique experimental paradigms. For example, changes of functional connectivity due to learning processes could be tracked in real time and the experiment be adjusted based on these observations. Furthermore, the connectivity estimates can be used for neurofeedback applications or the instantaneous inspection of measurement results. In this study, we present the implementation and evaluation of online sensor and source space functional connectivity estimation in the open-source software MNE Scan. Online capable implementations of several functional connectivity metrics were established in the Connectivity library within MNE-CPP and made available as a plugin in MNE Scan. Online capability was achieved by enforcing multithreading and high efficiency for all computations, so that repeated computations were avoided wherever possible, which allows for a major speed-up in the case of overlapping intervals. We present comprehensive performance evaluations of these implementations proving the online capability for the computation of large all-to-all functional connectivity networks. As a proof of principle, we demonstrate the feasibility of online functional connectivity estimation in the evaluation of somatosensory evoked brain activity

## 1 Introduction

To understand information processing in the human brain, identifying the active brain areas in resting state or in response to an external stimulus and discovering how these areas are connected and synchronized is highly desirable. Due to their unique time resolution in the millisecond range, electroencephalography (EEG) and magnetoencephalography (MEG) are noninvasive methods that are excellently suited for such investigations. Whereas the active brain areas can be identified by means of EEG/MEG source analysis, the estimation of functional connectivity in source or sensor space enables investigating the interplay between these brain areas. Functional connectivity exclusively relies on analyzing the statistical interdependency of activity in different brain regions without any prior assumptions about structural or causal relations between these regions. It is also excellently suited to observing transient and dynamic connectivity processes.

In practice, functional connectivity is represented in form of networks consisting of nodes and (weighted or binary) edges. The nodes can be defined as the measurement sensors or as source locations in the brain volume. The edge weights describe either the directed or undirected strength of the connectivity between the nodes and can be computed once connectivity metrics have been established. To date, a multitude of connectivity metrics has been proposed, which usually estimate functional connectivity between two or more nodes based on a statistical dependency between the time courses or phase or amplitude in certain frequency bands (Brookes et al., 2019). These metrics differ significantly both with regard to accuracy and stability but also computational effort (Bastos & Schoffelen, 2016;Sakkalis, 2011).

The analysis of the obtained connectivity networks allows to understand the activation of different brain networks which can, for example, be a result of different experimental conditions or training effects. Important tools in the analysis of these networks are methods and measures from graph theory, such as the computation of node degree, node strength, clusters, motifs, paths, and hubs (Bullmore & Sporns, 2009;Richiardi et al., 2013;Sporns, 2010;Stam, 2004).

A pitfall in the analysis of functional connectivity using EEG/MEG in sensor space is the occurrence of spurious connectivity, though the susceptibility to spurious connectivity differs strongly between connectivity measures. Common phenomena giving rise to spurious connectivity are the use of a common reference average in EEG, where the signal measured by the reference electrode can lead to spurious connectivity, or the effects of volume conduction, as the signal of any individual source is instantaneously picked up by all sensors (with varying magnitude). These problems can be mitigated by unmixing the sensor signals through source estimation and conducting functional connectivity analysis in the source space (Barzegaran & Knyazeva, 2017;Gross et al., 2001;Palva et al., 2018;Schoffelen & Gross, 2009;Silva Pereira et al., 2017). However, it has to be observed that inaccuracies in the source estimation, for example, due to simplified head models, can also influence functional connectivity estimates (Brunner et al., 2016;Cho et al., 2015;Mahjoory et al., 2017).Mahjoory et al. (2017)furthermore observed a considerable variability of estimated connectivity between different inverse approaches and recommend to verify obtained results using more than one inverse approach. Besides the erroneous computation of functional connectivity metrics, brain activity that cannot be measured with EEG/MEG can also influence the interpretation of functional connectivity.

EEG/MEG functional connectivity has been and is used in a multitude of studies to discover the brain mechanisms underlying diverse brain functions, such as motor learning during task execution (F. T. Sun et al., 2007), language processing (Gaudet et al., 2020;Hyder et al., 2021;Maess et al., 2016;Mamashli et al., 2019), and even during resting state (Albert et al., 2009;Brookes et al., 2011;Colclough et al., 2016;De Pasquale et al., 2010;Mackintosh et al., 2021). Correlations between functional connectivity and individual task performance were also found for other cognitive functions, including working memory (Hampson et al., 2006;Klados et al., 2019), visual perceptual learning (Lewis et al., 2009), statistical learning (Paraskevopoulos et al., 2017;Tóth et al., 2017), and face processing (Zhu et al., 2011). Functional connectivity networks have also been proposed as biomarkers for early disease prediction and prevention, for example, in schizophrenic patients (Hirvonen et al., 2017).

The brain response to median nerve stimulation at either the right or left wrist has been shown to include processing both in the primary (S1) and secondary (S2) somatosensory cortex as well as in the posterior parietal cortex (Hari & Forss, 1999).Simoes et al. (2003)demonstrated that this processing included phase locking between contralateral S1 and both contra- and ipsilateral S2 that was not time locked to the stimuli. Median nerve stimulation can be used to study healthy (Huang et al., 2000;Weisend et al., 2007;Wiesman et al., 2017) and abnormal (Huang et al., 2004;Ren et al., 2019) sensory processing. In this study, median nerve stimulation data are used to validate the new online functional connectivity pipeline.

The majority of these studies investigated “static” functional connectivity, that is, aimed to identify a single, static network of brain regions that is activated in response to a stimulus. This analysis usually ignored the temporal component of this activation. Recently, the analysis of “dynamic” functional connectivity has become more popular, which takes the temporal component of functional connectivity into account (O’Neill et al., 2018). This is not only of interest for evoked brain activity but especially also for resting-state/spontaneous brain activity. This analysis, for example, allows us to better understand MEG power modulations during resting state or the dynamics of functional networks during action control (Duprez et al., 2022;Liu et al., 2010).

The estimation of functional connectivity is usually performed offline, that is, after the experiment is concluded. However, scenarios are perceivable where online processing of EEG/MEG measurements, that is, the low-latency evaluation of data during the measurement, is desirable. A basic example would be “live” monitoring of the measurement outcomes to allow early intervention to fix possible problems, adjust the measurement time/number of recorded samples to achieve a certain signal-to-noise ratio (SNR), or to assess whether the experimental paradigm works as anticipated. But also innovative, dynamic experimental designs that directly depend on the measured brain states are conceivable. In clinical care, rapid analysis of normal or abnormal information processing, such as in the case of median nerve stimulation, or direct, intuitive insight into the patient’s brain state could lead to faster diagnosis and treatment.

To date, foci of online EEG/MEG processing have been brain–computer interface (BCI) and neurofeedback applications. Besides the development of basic sensor space operations, such as noise reduction and estimation of frequency band power, methods for the online estimation of cortical activity have also been implemented (Dinh et al., 2015,^2018^;Guarnieri et al., 2021). Even though the potential of functional connectivity analysis in online EEG applications such as BCI has been evaluated in numerous studies (Feng et al., 2020;Lee & Hsieh, 2014;Shamsi et al., 2021;Tabarelli et al., 2022;Torres et al., 2020), only a few implementations that allow at least a basic online estimation of EEG functional connectivity have been presented to date. This might be due to the computational complexity of the connectivity methods and/or the fact that the estimation of functional connectivity based on few samples, as it is common in online scenarios, is a complex task. Particularly the computation of all-to-all connectivity, that is, functional connectivity analysis without restrictive prior assumptions regarding the involved brain areas, in networks with a large number of nodes results in high computational complexity. Therefore, the connectivity estimation is often restricted to a small number of regions of interest (ROIs), or the number of sensors included is reduced in sensor space analysis.

In this paper, we describe the implementation of online functional connectivity estimation in the open-source software package MNE Scan, study the computational performance in large all-to-all connectivity networks of this implementation in scenarios using simulated and realistic data, and demonstrate the online capability of the obtained pipeline. The focus of the current implementation is on the determination of static functional connectivity of evoked brain activity. However, also dynamic functional connectivity in resting state can in principle be evaluated.

## 2 Methods

### 2.1 Connectivity metrics

The main novelty presented in this study is the implementation of online functional connectivity estimation for considerably large networks. Nine functional connectivity metrics were implemented, namely correlation (COR) (Schoffelen & Gross, 2009), cross-correlation (XCOR), coherency (COHY) (Bendat & Piersol, 2011), coherence (COH) (Bendat & Piersol, 2011), imaginary coherence (IMAGCOHY) (Nolte et al., 2004), phase-locking value (PLV) (Bruña et al., 2018), phase-lag index (PLI) (Stam et al., 2007), and its variations USPLI, WPLI, and DSW PLI (Vinck et al., 2011). The exact definitions of these metrics can be found in theSupplementary Material. The basic implementation of these metrics is described inSection 2.4and the resulting processing pipeline is described inSection 2.5.

Two of the nine metrics reflect relationships in the time domain (COR, XCOR), whereas the other seven are spectral metrics based on the frequency domain representations of the signals. Of course, there are considerable differences between these metrics concerning the computational complexity, which are usually of minor importance in offline data analysis but crucial in an online scenario. The effect of these differences on the computational effort in the online capable implementations of the connectivity metrics is evaluated inSection 3.1.

Besides the computational effort, the connectivity metrics also considerably differ with regard to the accuracy and stability of the obtained connectivity estimates. For offline analysis, a multitude of studies exploring the advantages and disadvantages of the different metrics have been performed (Barzegaran & Knyazeva, 2017;Bastos & Schoffelen, 2016;Sakkalis, 2011;Schoffelen & Gross, 2009;Vinck et al., 2011). The results of offline evaluations of functional connectivity metrics for the most part translate to the case of online connectivity analysis. However, due to the commonly low SNR achieved in online evaluations, the robustness of the metrics toward noise plays a crucial role here. The scenarios for which we evaluated the accuracy and robustness of the different functional connectivity metrics are described inSection 2.6and the results are presented inSection 3.2.

For reasons of conciseness, we only display the results for the metrics COH, IMAGCOH, PLI, and XCOR within the main manuscript, which stand exemplary for the three main types of metrics considered (time domain, spectral taking phase of the signal into account, spectral using imaginary part). The results for all metrics can be found in Section 2 of theSupplementary Material.

### 2.2 MNE-CPP

The MNE-CPP project provides a framework to develop and build online as well as offline analysis software for electrophysiological data. The core development is done solely in C++ (CPP) and is, therefore, targeting more experienced programmers, whereas the resulting stand-alone applications can be easily used by researchers and clinical personnel without coding background. MNE-CPP is based on a two-layer architecture. The library layer provides the core functionalities, which can be used for the development of stand-alone applications and is loaded during runtime. The stand-alone applications can either be realized with a graphical user interface (GUI) or a command line interface (CLI). Furthermore, a multitude of examples and tests are implemented on the application layer of MNE-CPP.

To maximize cross-platform compatibility and simplify project maintenance, MNE-CPP makes use of only one external dependency, namely the Qt framework (https://www.qt.io). Furthermore, it uses the Eigen library via a so-called clone-and-own approach (https://eigen.tuxfamily.org). Qt provides tools for GUI creation, whereas Eigen provides tools for linear algebra computations. Minimizing the use of software of unknown provenance (SOUP) is favorable when developing medical software applications that have to meet regulatory requirements: each third-party dependency must be tracked, and its development life cycle must be auditable. Furthermore, all dependencies are able to compile on multiple platforms and devices for cross-platform capability in order to develop stand-alone applications on Windows, macOS, and Linux (Esch et al., 2018). MNE-CPP is open-source BSD licensed (clause 3) and accessible viahttps://mne-cpp.github.io/andhttps://github.com/mne-tools/mne-cpp.

Further details regarding the architecture of MNE-CPP can be found in the reference publication (Esch et al., 2019) and onhttps://mne-cpp.github.io/.

### 2.3 MNE Scan

MNE Scan is a tool developed within the MNE-CPP software project to acquire data from MEG/EEG devices and process the resulting data streams online. MNE Scan is designed as a plug-in-based software, meaning that acquisition and processing tasks are developed as individual units. This ensures a modular software architecture, which can easily be extended by new plug-ins with support for new hardware devices or analysis methods. MNE Scan distinguishes two kinds of plug-ins: Algorithm and Sensor plug-ins. Sensor plug-ins can only have output connectors, whereas Algorithm plug-ins can have both input and output connectors. The workflow in MNE Scan follows a pipeline approach where the user can select and connect Sensor and subsequent Algorithm plug-ins.

Currently, there are nine Sensor plug-ins, which offer connections to MEG (MEGIN, BabyMEG) and EEG (TMSI Refa, EEGoSports, gTecUSB, Natus, BrainAmp, and Lab Streaming Layer/LSL) devices. It is also possible to stream prerecorded data from disk in order to mimic a measurement session and test the online processing. The available Algorithm plug-ins include online capable implementations of, for example, temporal filtering, signal space projection (SSP) (Uusitalo & Ilmoniemi, 1997), SPHARA (Graichen et al., 2015), trial averaging, source estimation, and—as presented in this study—functional connectivity estimation. Measurement data can be stored in the fif format, which is the standard for all MNE tools and can be imported by most toolboxes. Furthermore, the data can be converted to the BIDS format subsequently (https://mne.tools/mne-bids/stable/index.html). Further details regarding the architecture of MNE Scan can be found in the reference publication (Esch et al., 2018) and onhttps://mne-cpp.github.io/pages/documentation/scan.html.

Besides MNE Scan, various open-source toolboxes for online EEG/MEG processing have been developed. A comparison of the basic features and requirements for a selection of these toolboxes is shown inTable 1. Whereas all of these toolboxes include the basic functionalities necessary for EEG/MEG processing and BCI applications, each of them has its own unique features. A complete overview and comparison of the features of these toolboxes are not possible within the scope of this manuscript. However, MNE Scan’s online EEG/MEG source analysis and online large-network EEG/MEG functional connectivity analysis in source and sensor space features are—to the best of our knowledge—unique among these toolboxes.

**Table 1.** Overview of open-source toolboxes for online EEG/MEG analysis.

Toolbox	MNE Scan	MNE-Python	OpenVibe	OpenBCI	FieldTrip	BCI2000	BCILAB
Dependencies	1	15	7	1	1	4	1
Modalities	MEG, EEG	MEG, EEG, sEEG, ECoG	EEG, (MEG ^a^ )	EEG	MEG, EEG, fMRI	MEG, EEG	EEG
Platforms	Win, MacOS, Linux	Win, MacOS, Linux	Win, Linux	Win, MacOS, Linux	Win, MacOS, Linux	Win, MacOS, Linux	Win, MacOS, Linux
Free of charge ^b^	✓	✓	✓	✓			
Reference	Esch et al. (2018)	Gramfort et al. (2013)	Renard et al. (2010)		Oostenveld et al. (2011)	Schalk et al. (2004)	Kothe and Makeig (2013)

Adapted fromEsch et al. (2018).

a Not part of stable release.

b Considering additionally required licenses, for example, Matlab.

### 2.4 Implementation of functional connectivity metrics

The functional connectivity metrics were implemented in the new Connectivity library as part of the MNE-CPP library layer. This library includes routines to calculate functional connectivity metrics, a container to store the resulting networks, and an Application Programming Interface (API). All features were implemented with the goal of achieving a performance that enables the online capability of the resulting applications.

The library was designed to be as generic as possible in order to not limit its functionality to only a set of specific use cases or measurement modalities. It should further be extensible to include new connectivity metrics in the future. The library can be divided into three groups of classes, namely the API classes, the actual computational classes for each functional connectivity metric, and the network (data) container classes (seeFig. 1). Here, connectivity networks are first calculated for each trial and the resulting connectivity estimates for each trial are subsequently averaged. Thereby, also phase locking between brain regions that is not phase locked to the stimulus can be detected. Spontaneous or resting-state data can be processed by the library in the form of data blocks and handled as if they were multiple trials. This allows to apply a sliding window approach, which is a common approach to study dynamic functional connectivity (O’Neill et al., 2018).

**Fig. 1.**
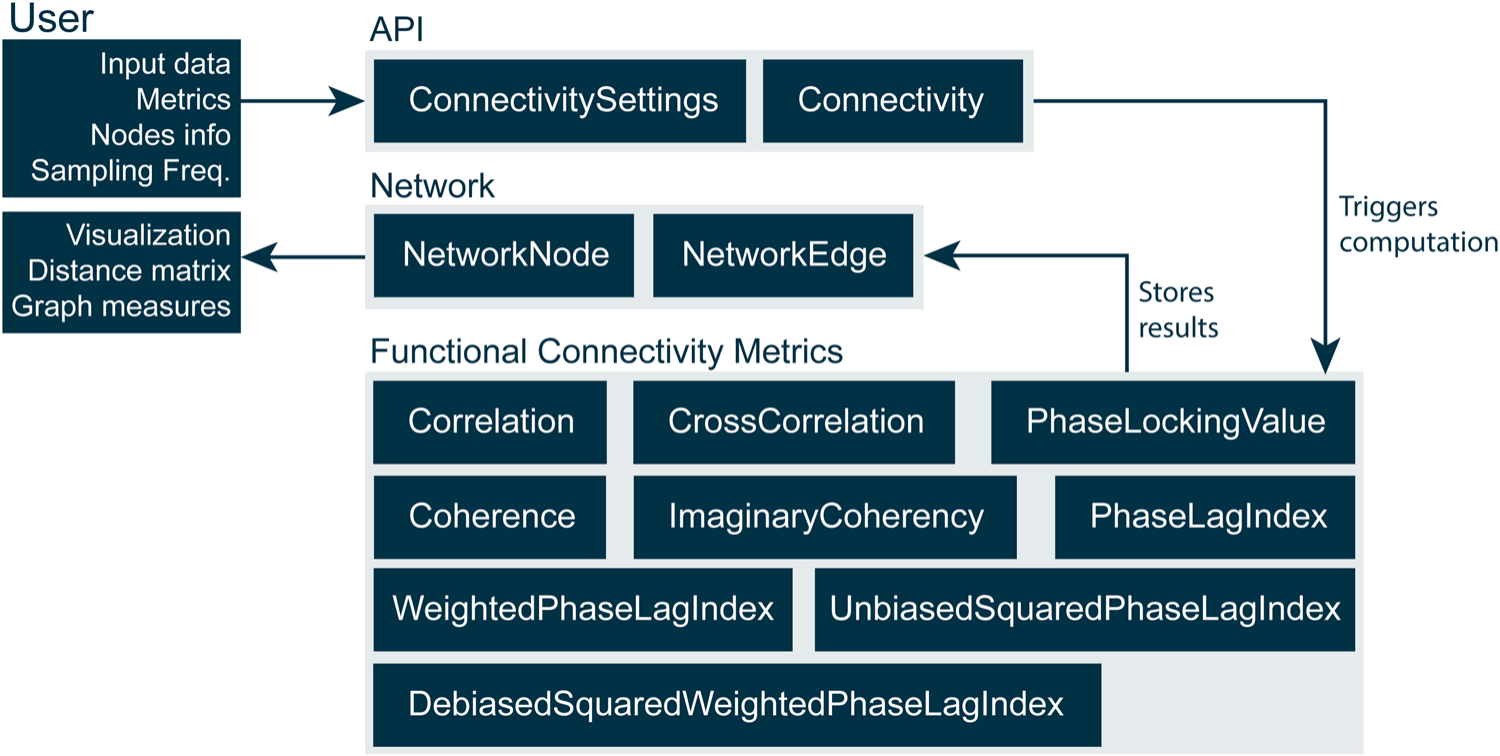
The three components of MNE-CPP’s connectivity library. The Connectivity class can be used to trigger the actual computations provided by the functional connectivity metric classes. The final result is provided back to the user in form of a Network class, which can then be used to analyze the network with the help of visualization tools, graph measures, etc.

In the implementation of the functional connectivity metrics, special emphasis was given to three key aspects to ensure the efficient implementation of the Connectivity library. First, multithreading was employed to process each trial in parallel. Second, the parallel functions were optimized with respect to efficient usage of iterative steps in loops, data storing, etc. Third, reusable data are carried over in order to ease the computational burden for subsequent iterations.

The COR metric is realized as a simple dot product and division by the number of samples. The XCOR metric utilizes an FFT convolution, which consists of the creation of the signal spectra, the multiplication of these spectra, and the transformation of the product back to the time domain. Subsequently, the highest correlation value and its index are returned.

All phase synchronization-based metrics are calculated in a very similar manner exploiting a running average to avoid unnecessary repetitions of computations, as we will explain in the example of the COH metric. For each trial, the frequency spectrum, cross-spectral density (CSD), and power spectrum density (PSD) are calculated. If these values have been calculated in a previous step for this trial, the computation will be skipped and the stored values will be used. Besides the CSD and PSD per trial, the sums of CSD and PSD over all trials are also directly computed, which saves one iteration step for computing the average over all trials after the computation of CSD and PSD for all trials is finished. Subsequently, these sums are used to perform the actual computation of the COH metric, where the sensors and their corresponding contributions are processed in parallel. Since COH is an undirected metric, only one half of the edge weights need to be calculated. This procedure is applied for all implementations of the phase synchronization-based metrics, adapted to the respective definition of each metric.

The numerous necessary FFT computations are realized using efficient external libraries, which are distributed with the Eigen library, so that this does not add any additional external dependencies. The Connectivity library’s FFT backend supports the Keep It Simple Stupid Fast Fourier Transform (KISSFFT,https://github.com/mborgerding/kissfft) and Fastest Fourier Transform in the West (FFTW,http://www.fftw.org).

Further details regarding the underlying architecture and the implementation of the Connectivity library can be found onhttps://mne-cpp.github.io/pages/development/api_connectivity.html.

### 2.5 Online functional connectivity pipeline

The functionalities of the connectivity library in combination with newly implemented tools for visualization, the Disp3D library, were used to implement a new online connectivity pipeline in MNE Scan. The plug-ins included in the pipeline depend on the scenario being investigated. Four possible modes reflecting different scenarios are depicted inFigure 2.

**Fig. 2.**
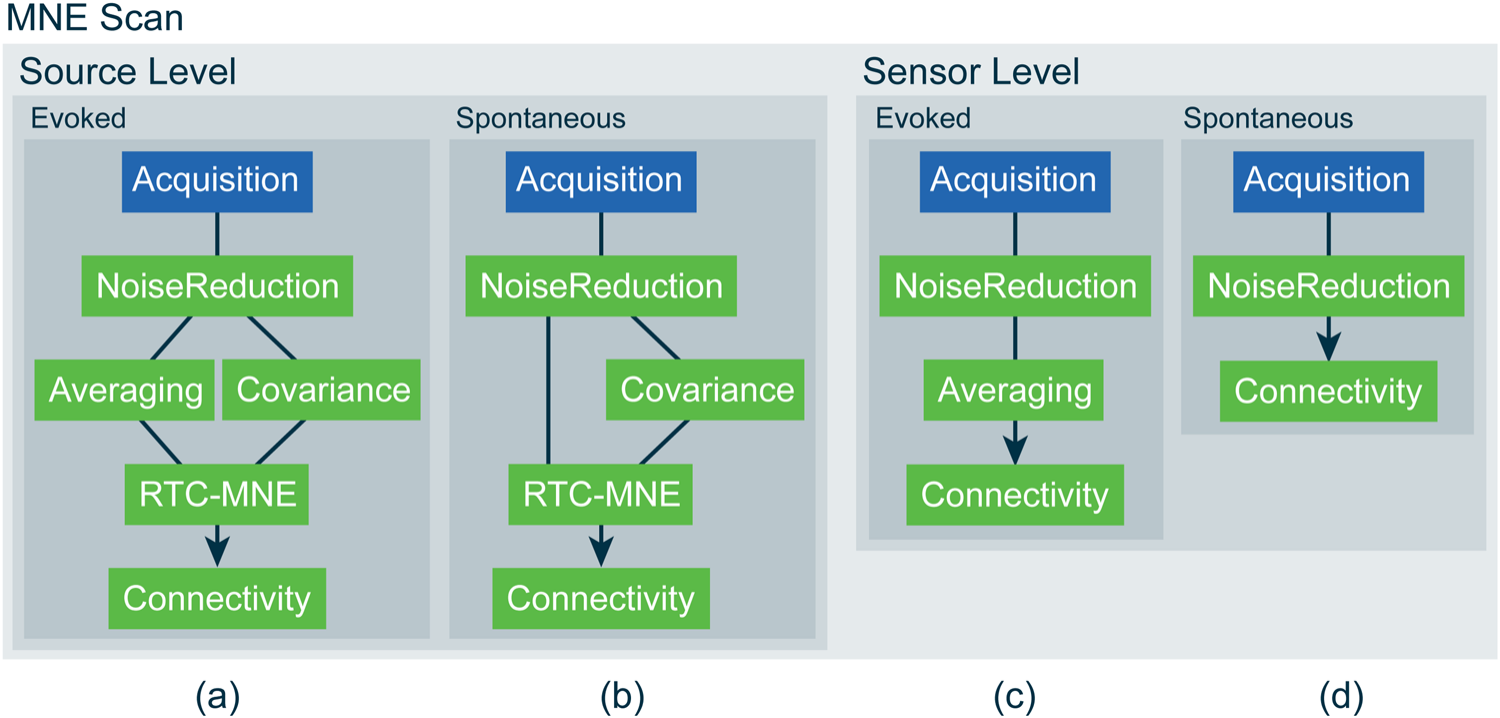
Sketch of the implemented online functional connectivity pipelines in MNE Scan. Four different modes can process source- (a, b) and sensor-level (c, d) data from measurements of either evoked response experiments (a, c) or spontaneous activity (b, d). The sensor (acquisition) plug-in is depicted in blue, whereas the algorithm (processing) plug-ins are shown in green.

Functional connectivity networks can be estimated and visualized based on sensor- and source-level data. For evoked response experiments, the computation can be done with data blocks obtained from the Averaging plug-in based on an external trigger, as explained in more detail later, and for continuous measurements of, for example, resting-state data, the computation can be performed directly based on the data blocks obtained from a Sensor plug-in.

The possible choices for the acquisition plug-ins correspond to the sensor plug-ins described inSection 2.2; the implementation of additional sensor plug-ins based on a self-development kit (SDK) provided by the respective manufacturer is commonly straightforward following the architecture of the existing sensor plug-ins.

The Noise Reduction plug-in is implemented with the goal of also performing the necessary data preprocessing within online evaluation pipelines. It includes temporal filtering, SSP and synthetic gradiometers (L. Sun et al., 2018), and SPHARA methods (Graichen et al., 2015) and has been previously described inEsch et al. (2018).

For temporal filtering, a design tool can be used to create finite impulse response (FIR) filters on the fly during the ongoing measurement. The design process is controlled by the cutoff frequencies, transition bandwidth, filter method, and filter taps. Low-, high-, and band-pass filters are supported. All implemented filters provide a linear phase response and hence a constant group delay. This property is particularly important when dealing with functional connectivity metrics based on phase synchronization. The actual filter operation is realized with an FFT convolution and all channels are processed in parallel. Since the data are received in the form of blocks, the overlap-add method was implemented to cope with the delay introduced by filtering.

The Averaging plug-in processes trigger channels present in the incoming data stream in order to detect events (Dinh et al., 2015;Esch et al., 2018). The distinction between multiple event types is supported to allow for experiments with more than one stimulus type. If desired, a moving average is employed to average the trials, produce the resulting evoked responses for each event type, and make them available for subsequent connected plug-ins.

The Covariance plug-in computes the covariance matrix every time the amount of data specified by the user has been gathered. The resulting covariance matrix is forwarded to the Real-Time Clustered Minimum Norm Estimate (RTC-MNE) plug-in. This process accounts for changing noise levels over the course of the measurement session.Dinh et al. (2015)propose a source space reduction through a region-wise clustering based on the Destrieux brain atlas (Destrieux et al., 2010), which is provided by the Freesurfer toolbox. Based on the clustered forward solution, the inverse operator is calculated every time a new covariance estimate arrives and is then used to map the sensor-level data to the source space for each data block. In comparison with the prior work ofDinh et al. (2015), the RTC-MNE plug-in was improved in order to also handle raw data streams (nonaveraged data), enabling the analysis of spontaneous data.

Finally, the Connectivity plug-in provides access to the functional connectivity algorithms implemented in the Connectivity library. To allow for a variety of different online functional connectivity pipelines, it accepts three different types of input data. First, data blocks of, for example, spontaneous brain activity streamed directly from an acquisition plug-in, possibly after noise reduction. Second, evoked response data forwarded by the Averaging plug-in. Third, source-level data, for example, generated by the RTC-MNE plug-in. In the case of evoked data, the Averaging plug-in is only used to cut out the data segment of interest. This means if functional connectivity based on an evoked response experiment is to be investigated, the number of trials in the Averaging plug-in is always set to one. The actual averaging for the desired number of trials is performed in the Connectivity plug-in after the functional connectivity estimate has been calculated for each trial. The resulting network is sent to the online visualization display and all subsequent connected plug-ins. The online visualization display, which is implemented using the Disp3D library, enables a 3D visualization of both source- and sensor-level data. Among other parameters, a higher or lower threshold for the truncation of the connectivity network can be selected to avoid cluttered visualizations or show also weaker connections, respectively.

An overview of the most important parameters for the different plug-ins used in the connectivity pipeline is presented inTable 2. Parameters that can be adjusted during runtime (which is the majority of parameters) can be accessed via the*PluginControlWidgets*embedded in the*QUICK CTRL*menu. Parameters that can only be adjusted before measurement start can be accessed via the*SetupWidgets*in the main program window.

**Table 2.** Overview of (selected) parameters for plugins used in Connectivity pipelines.

Plugin	Submenu	Parameters	Options/range	Comments
**Acquisition**		Sampling Frequency (Hz) ^a^	*Depending on measurement device*	Separate Acquisition plugin for each manufacturer
		Block size (samples) ^a^	*Depending on measurement device*	
**Forward** ^b^		Brain Atlas Directory	*Path to brain atlas*	
		Cluster Forward Solution	yes/no	
		Number of Sources		
		Compute EEG/MEG Solution	yes/no	
		Fixed Orientation ^a^	yes/no	Use normal constraint in source analysis
		Compute in MRI Space ^a^	yes/no	
		Filter Source Space ^a^	0–100 mm	Remove source positions too close to skull
**Noise Reduction**	SSP	*Depending on measurement device and provided data*		
	Compensators	*Depending on measurement device and provided data*		For example, CTF compensation
	Filter	Design method	Tschebyscheff, Cosine	
		Channel type	EEG, MEG, ECG, EOG	
		Range	0–999 Hz	
		Transition band	0–999 Hz	
		Filter taps	16–4,096	
	Sphara	System	BabyMEG, VectorView, EEG	
		Inner layer/Outer layer	1–270, 1–105	For “single-layered” measurement setups (e.g., EEG, CTF),
		only *Inner layer* parameter, for “two-layered” setups (e.g., MEGIN), *Inner layer* (gradiometers) and *Outer layer* (magnetometers).		
**Averaging**	Averaging	Stimulus Channel	*Depending on measurement device*	
		Number of trials	1–1,000	Set to 1 for evoked connectivity measurement
		Pre-/Poststimulus	-10,000–0 ms, 0–10,000 ms	
	Baseline	Min/max	-10,000–0 ms, -10,000–0 ms	
	Artifact	Threshold, Factor	0–100,000, 10 ^−10,000^ –10 ^0^	Unit depending on channel type; threshold and factor are multiplied
**Covariance**		Number of Samples	600–36,000	
**RTC-MNE**		Method	MNE, dSPM, sLORETA	
		Trigger type	*Depending on measurement device*	
		Time point	1–10,000	Selects time point of visualization
**Connectivity**		Metric	COR, COH, IMAGCOH, XCOR, PLI, DSWPLI, USPLI, WPLI, PLV	
		Window type	Hanning, Ones	
		Number of trials	1–1,000	
		Frequency band	0–10,000	Limited by recording frequency
**3D View** ^b^		Colormap min/middle/max	0–max, 0–max, 0–max	Adjustment dialog opens upon left click on threshold values Colorbar min also corresponds to lower threshold

a Can only be adjusted in*SetupWidget*before to measurement start.

b Not all options listed.

### 2.6 Evaluation

Two different scenarios were used for evaluation, one based on simulated and another one based on actual measurement data. The computations were performed on a Linux CentOS 7.6 desktop setup including an Intel Xeon X5660 CPU with 12 physical (24 logical) cores running at 2.8 GHz, 47GB RAM, and an NVIDIA Quadro 4000 graphics card.

For an initial verification, each spectral functional connectivity metric was tested against its implementation in the MNE-Python toolbox (Gramfort et al., 2013). COR and XCOR were tested against their Matlab equivalents. The routines of MNE-CPP, MNE-Python, and Matlab were used to process the same data and compare the final results. No differences were found.

#### 2.6.1 Test scenario: Simulation

**Fig. 3.**
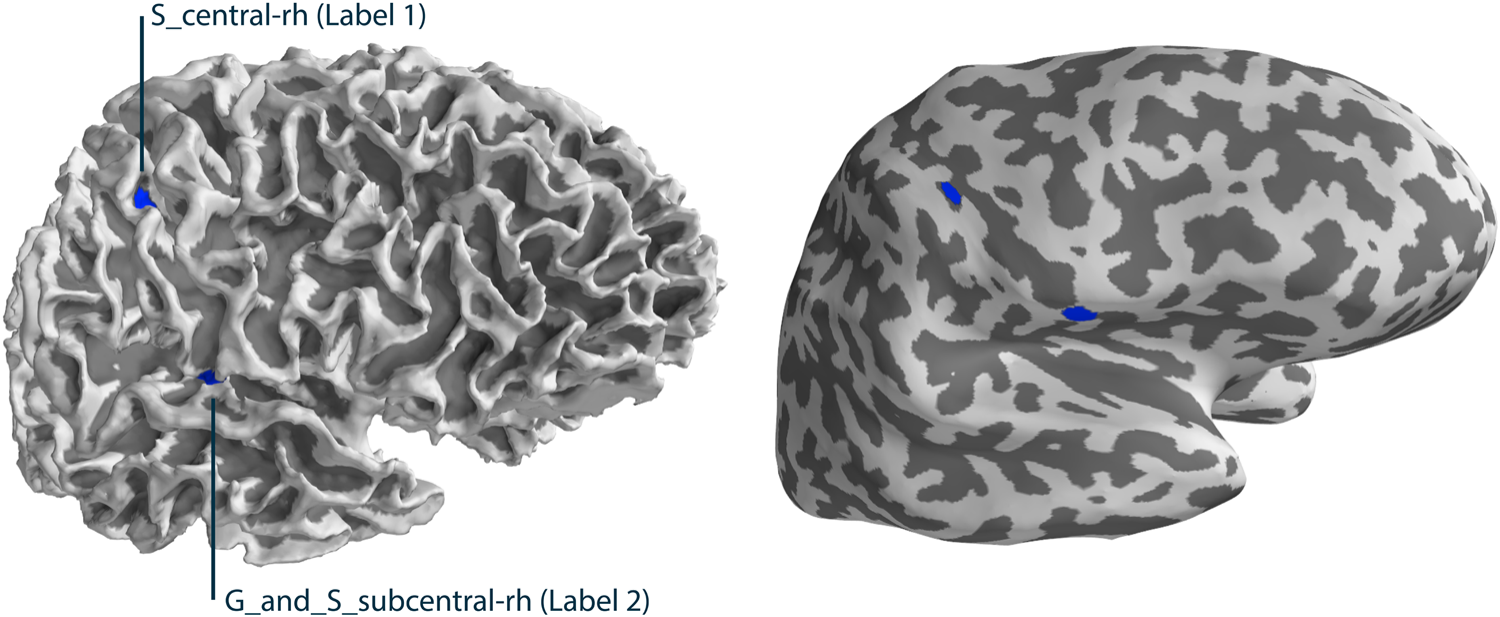
Labels defined from the S_central-rh and G_and_S_subcentral-rh patches.

In a first test scenario, a simulation was performed so that the ground truth for source locations and connectivity is known and can be compared with the reconstructions. The simulation was created with the MNE-Python toolbox. Two labels were defined with a 5 mm diameter inside the S_central-rh and G_and_S_subcentral-rh patches extracted from the Destrieux brain atlas (Fig. 3). Label 1 included two source dipoles which were both driven with an 18 Hz sinusoid at 10 nAm and Label 2 included four source dipoles which were driven with an 18 Hz cosine at 10 nAm each so that we have a phase difference of exactly 90° between Label 1 and Label 2. Based on an existing three-layer BEM forward solution, the simulated source signals were projected to MEG sensors. The used forward solution was extracted from the sample_audvis-meg-oct-6-fwd.fif file included in the MNE-Sample-Data-Set (https://mne-cpp.github.io/pages/download/sample_data.html). Gaussian noise was added to the signals resulting in an SNR of 11.85 dB averaged over all MEG channels. For the source space connectivity estimation, RTC-MNE was chosen as the inverse method based on a reduced source space of 243 sources with the regularization parameter set to*λ*= 1*/SNR*^2^(Dinh et al., 2015). The duration of the simulated signal was set to 160 ms.

#### 2.6.2 Test scenario: Measurement

To simulate a real measurement session, the FiffSimulator plug-in was used to stream data from the prerecorded MIND data set. These data were recorded at the MIND Institute and its partner sites and internal review board approval was obtained at each of the sites (Weisend et al., 2007,^2022^). The paradigm was chosen to be a right-hand median nerve stimulation. A source-level pipeline based on evoked data was chosen, as depicted inFigure 2a. The pipeline included a noise reduction step with activated temporal filtering and SSPs. For comparison, the data were analyzed offline as well.

Right-hand median nerve stimulations of subject mind010 were extracted and filtered with a 2 Hz cutoff high-pass filter. Trials ranging from -100 to 400 ms with an EOG higher than 150 mV, excluding the stimulus artifact, were rejected. This left 200 trials remaining for processing. Each trial was baseline corrected with the prestimulus interval. The three-layer BEM forward model was clustered based on the Destrieux atlas (aparc.a2005s) (Fischl et al., 2004). The cluster size was set to 40 which reduced the source space from 7,423 to 265 active sources. The resulting source space is depicted inFigure 4.

**Fig. 4.**
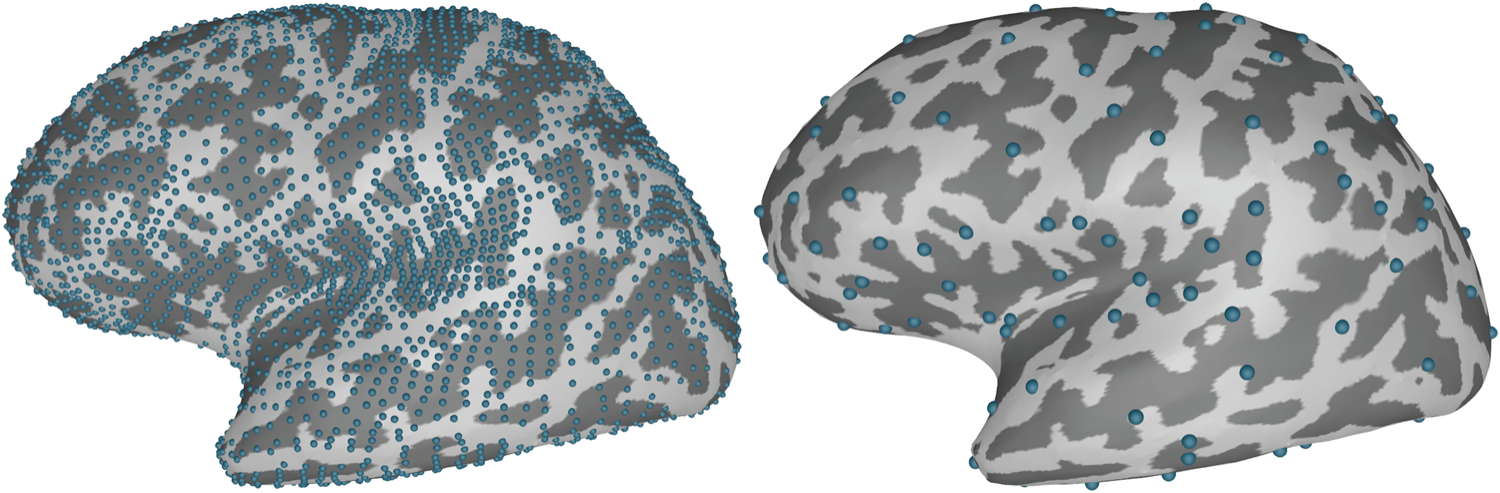
The source space for subject*mind010*before and after clustering with the RTC-MNE method based on the*aparc.a2005s*annotation information.

## 3 Results

Besides the evaluation of the accuracy of the functional connectivity estimation in the simulated and realistic data scenario introduced inSection 2.6, we also performed an in-depth evaluation of the computational performance of the Connectivity library. The results of this evaluation are presented first of all in this section.

### 3.1 Performance

Each functional connectivity metric was evaluated with different number of channels and trials and with different window sizes. Each computation was repeated five times; the performance data reported are averages over the five repetitions. The analysis steps included the actual metric calculation and creation of the final network data container. The FFT length was set to 600 bins, matching the sampling frequency of 600 Hz to result in a frequency resolution of 1 Hz. The FFTW was chosen as the FFT backend and the frequency band was chosen from 8 to 12 Hz (4 bins). The storage mode was turned off, meaning the connectivity estimates for each trial were recalculated for each performance run.

The performance values (computing time) determined for each functional connectivity metric and one trial over various window sizes as well as computed nodes are presented inFigure 5. The performance values determined for each functional connectivity metric and 1,000-sample window size over various number of trials as well as computed nodes are presented inFigure 6.

**Fig. 5.**
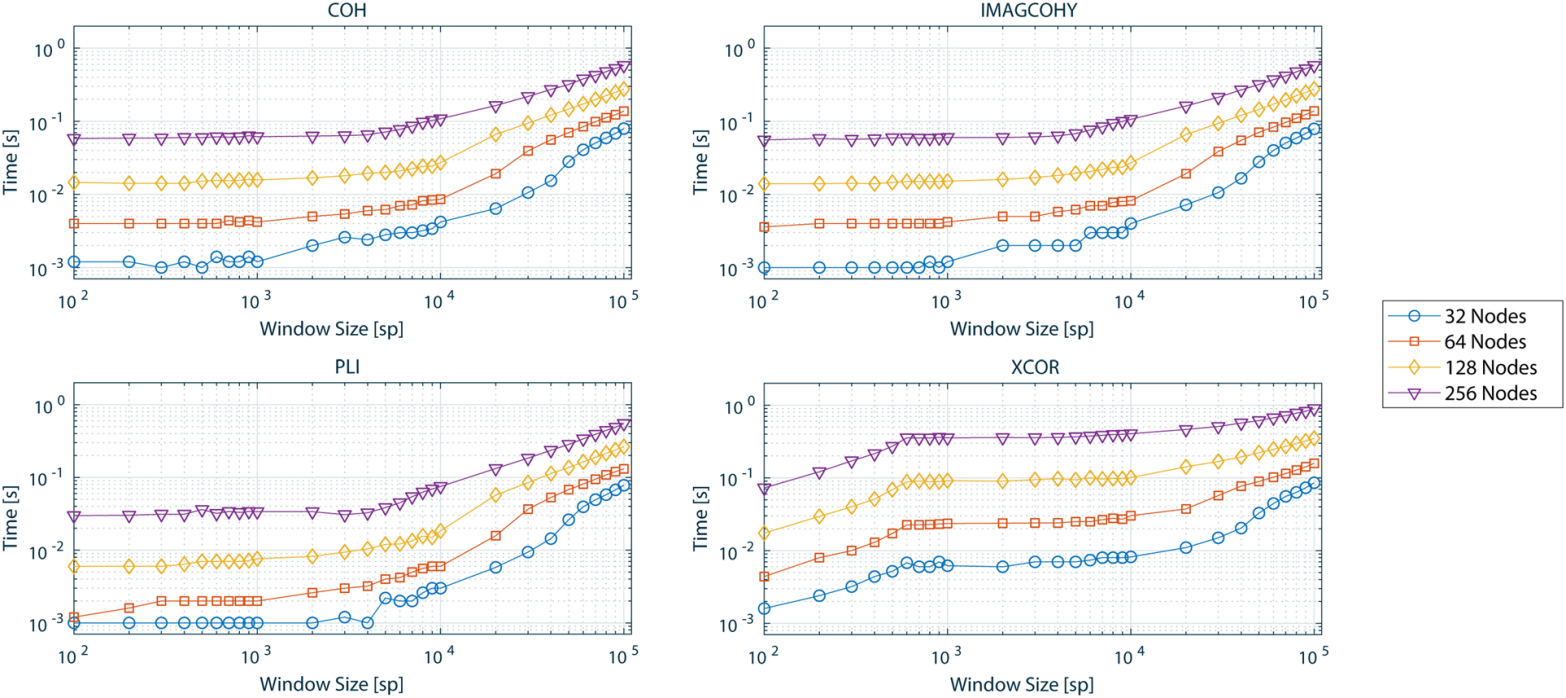
Computational timing values in seconds for one trial and different window sizes in samples (sp) as well as the number of computed nodes (y-axis in logarithmic scale) for COH, IMAGCOHY, PLI, and XCOR metrics. Computations were averaged over five repetitions. Results for all metrics can be found in Figure SM 1 in theSupplementary Material.

**Fig. 6.**
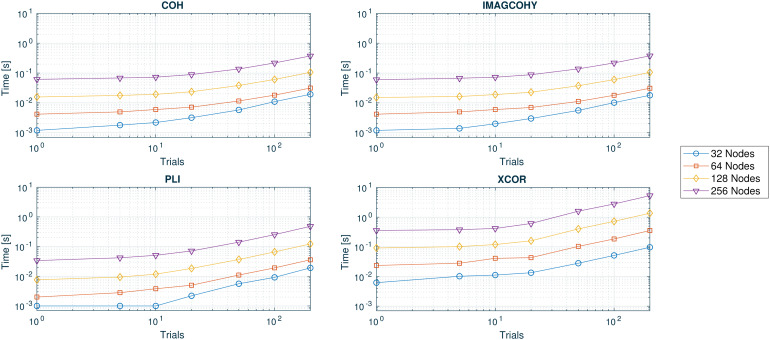
Computational timing values in seconds for 1,000 sp and different number of trials as well as computed nodes (y-axis in logarithmic scale) for COH, IMAGCOHY, PLI, and XCOR metrics. Computations were averaged over five repetitions. Results for all metrics can be found in Figure SM 2 in theSupplementary Material.

The timing results as a function of the number of samples show similar timing values that are approximately constant for all frequency-based metrics up to 5,000 sp to 6,000 sp (Fig. 5), where PLI is less computationally costly than COH and IMAGCOHY. The XCOR timing values are higher compared with every other metric, also when evaluating the timing as a function of the number of trials. The COR metric outperforms all other metrics (Fig. SM 1 in theSupplementary Material).

Also when run on multiple trials, the frequency-based metrics show similar timing values, again with PLI being slightly more efficient than COH and IMAGCOHY for small number of trials (Fig. 6; Fig. SM 2 in theSupplementary Material). Again, the XCOR timing values are higher than for the frequency-based metrics.

In both test settings, the timing values increased with the number of network nodes for all metrics, where the increase is about linear for small window sizes.

### 3.2 Evaluation

#### 3.2.1 Simulation

**Fig. 7.**
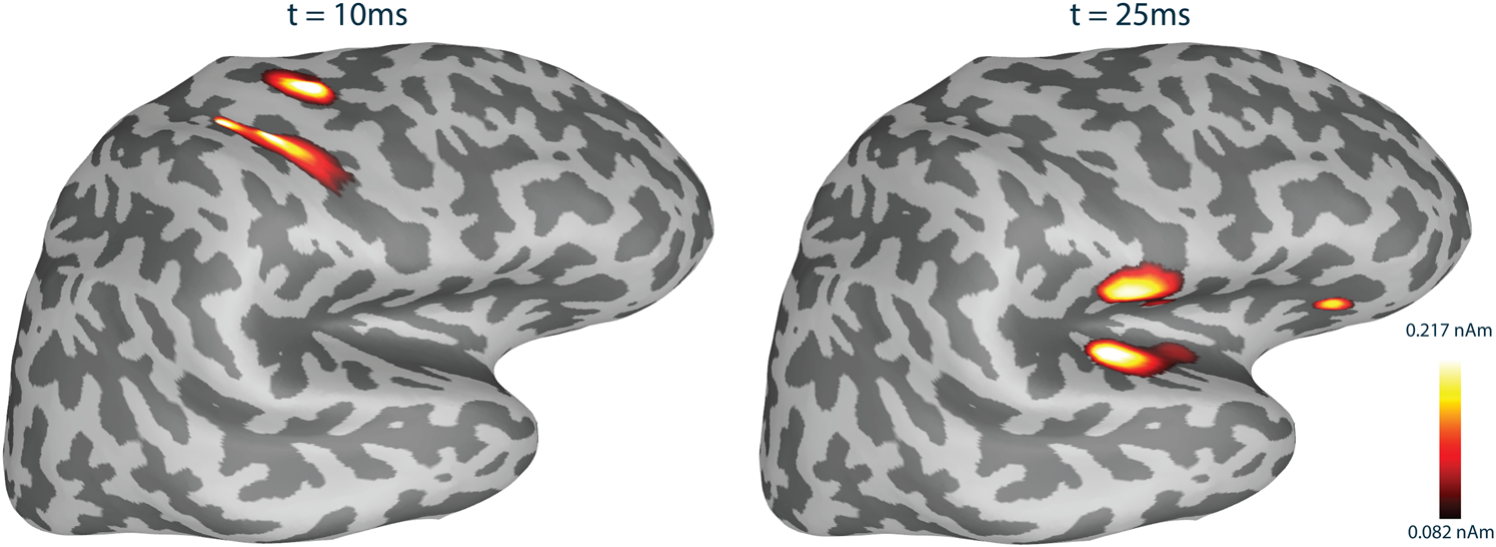
Source activity reconstructed with the RTC-MNE method based on simulated data. The processed average included 200 trials. Note that the absolute value of the source activation was plotted.

Functional connectivity metrics were computed for the RTC-MNE source reconstructed time courses for 200 trials. In order to be comparable with the steps performed within the pipeline for real data, all trials were baseline corrected with the data segment -50 to 0 ms.Figure 7shows the source reconstruction using the RTC-MNE method at two different time points.

For the connectivity calculation, each trial was cropped from 0 to 160 ms. The sampling rate of the input data was 600 Hz. Frequency bins 0 to 50, which corresponded to 0 to 50 Hz, were averaged to calculate functional connectivity networks. No ROI selection was performed. Instead, all-to-all connectivity networks of the 243 sources were computed. The storage mode of the Connectivity library was turned on, meaning subsequent metric computations used already calculated and stored intermediate data. All networks were normalized based on their maximum edge weight.

Due to the phase difference of exactly 90° between the 2 simulated source time courses (seeSection 2.6.1), all true connectivities for the frequency domain connectivity metrics are exactly 1. However, a perfect reconstruction of the simulated connectivity cannot be expected due to, for example, the added noise and imperfect source reconstructions (Bastos & Schoffelen, 2016). Furthermore, spurious connectivity between neighboring activated sources has to be expected for those connectivity metrics that do not suppress instantaneous connectivity.

**Fig. 8.**
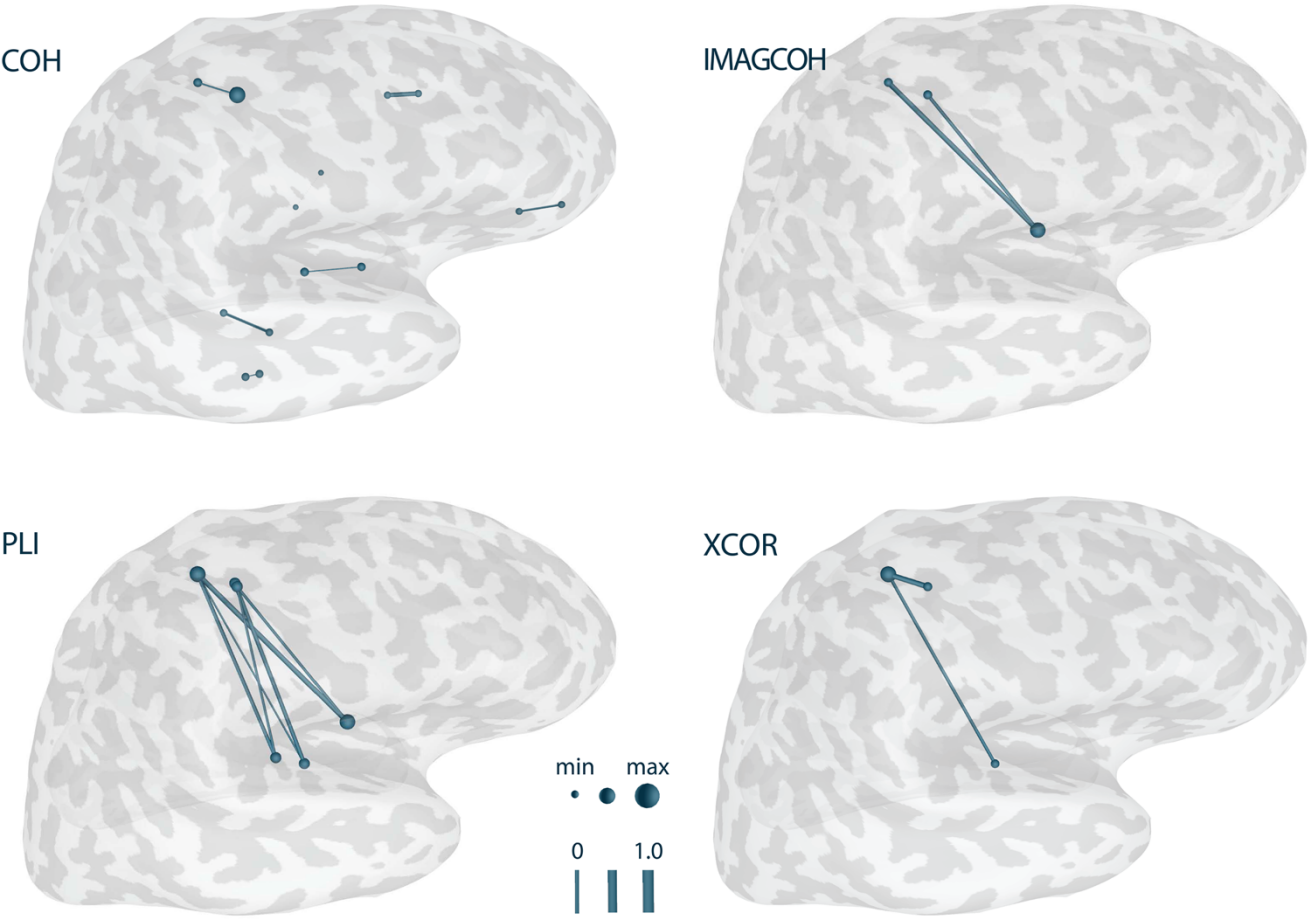
Functional connectivity networks for different metrics based on simulated data. The RTC-MNE method was used to compute the source activity. The number of trials was 200. Network nodes are plotted as spheres and edges are represented as tubes connecting the nodes. Edge strength and node degree are represented by their diameter. Only the edges representing the strongest 5% of connections are plotted. Note that the nodes’ sphere diameters are normalized by the node with the maximal value in the thresholded network. Results for all metrics can be found in Figure SM 3 in theSupplementary Material.

Figure 8shows the visualization of the COH, IMAGCOHY, PLI, and XCOR functional connectivity metrics with the edges representing the strongest 5% of connections. For each metric and corresponding network, the maximum edge weight was identified and used to normalize all edge weights. It can be observed that the metrics based on the imaginary valued part of the CSD, that is, IMAGCOHY and PLI, were able to detect the functional connection between the two simulated activation areas L1 and L2. For the COH metric, all of the strongest 5% of connections were very short-range (instantaneous) connectivity. The true connectivity might only become visible when filtering out this spurious connectivity, for example, by suppressing very short-range connectivity or defining ROIs based on a priori assumptions. These trends also hold true for other metrics based on the imaginary part of coherency or including the phase of the signal, respectively, such as WPLI and DSWPLI on the one hand or PLV on the other hand (see Figure SM 3 in theSupplementary Material). Also, XCOR seemed to be able to detect some functional connection between L1 and the temporal lobe activity spread from L2.

The relationship between the connectivity weights and the number of trials is presented inFigure 9. For each metric, the 20 strongest edges at 200 trials were identified (Fig. 8). The exact same edges and their averaged weights were computed for different number of trials subsequently. This information gives insight into how the strength of the connections detected in the final network at 200 trials evolved with an increasing number of trials. However, it has to be observed that the convergence of the connection strength with the increasing number of trials does not reflect the correctness of the results; it merely reflects how quickly the detected stable network is established. It can be observed that all metrics except for COR and IMAGCOHY started to stabilize around approximately 20–40 trials. The averaged IMAGCOHY weights started to stabilize beginning at approximately 60 trials.

**Fig. 9.**
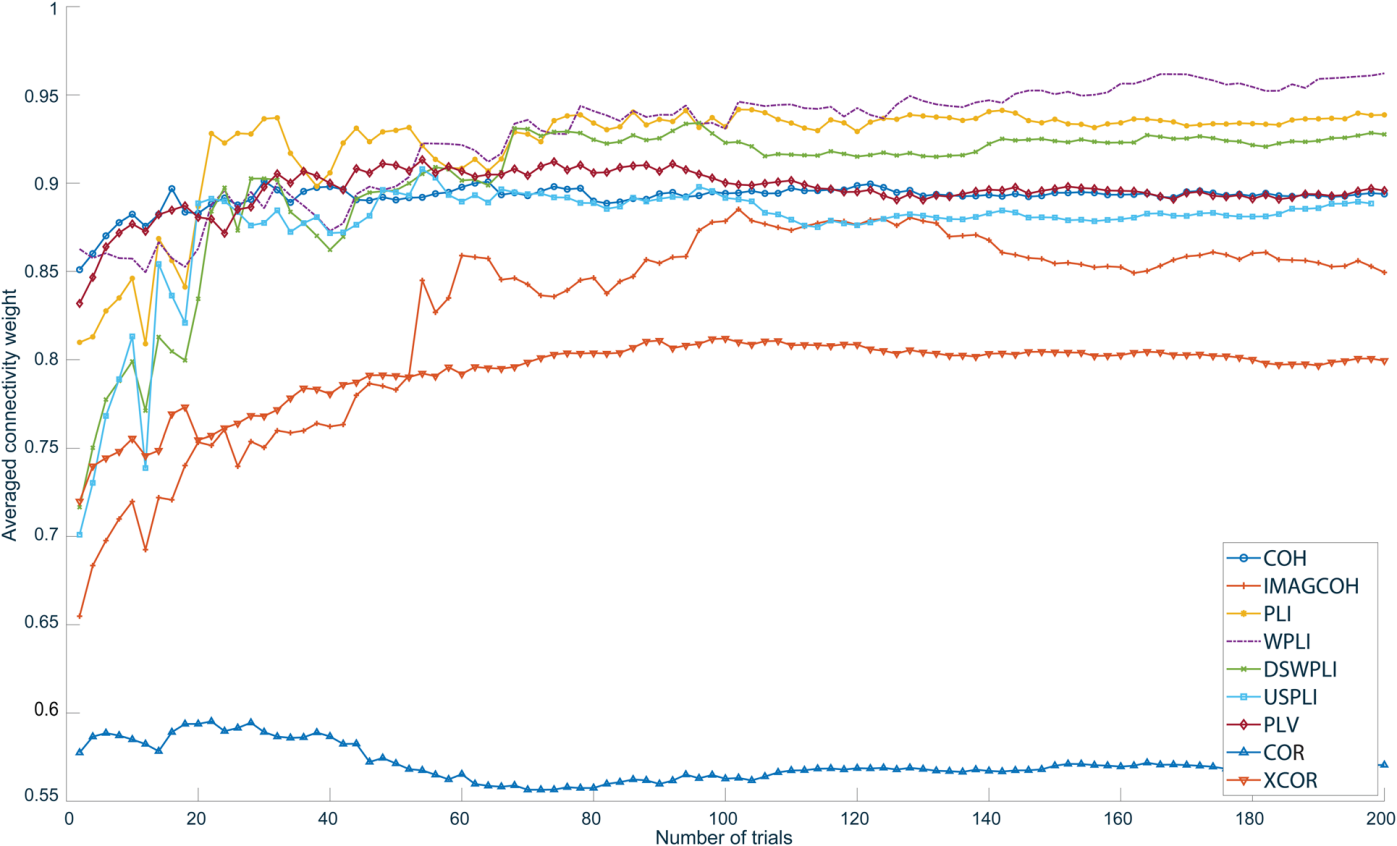
The relationship between the final networks at 200 trials and networks calculated at different number of trials. The networks were estimated for all metrics based on simulated data. Observe the cropped y-axis.

#### 3.2.2 Application/proof-of-principle

**Fig. 10.**
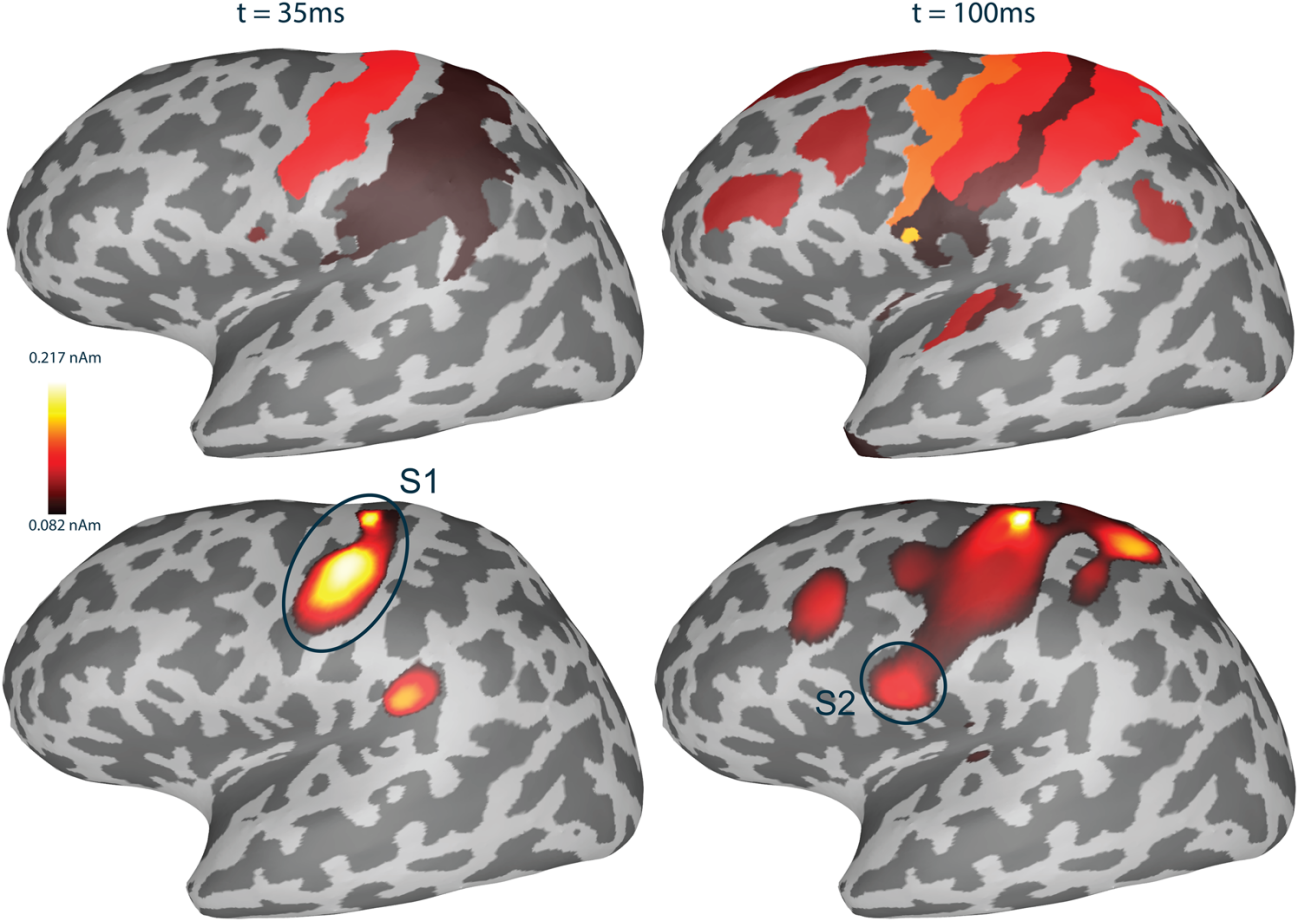
RTC-MNE results for a 200-trial average and 2 time instances based on right-hand median nerve stimulation. The S1 and S2 areas are indicated by blue circles. The upper row presents the patch-based visualization, whereas the lower row shows the surface-constrained interpolation visualization. Note that the absolute value of the source activation was plotted.

RTC-MNE source estimation was performed for the 200 trials obtained as described inSection 2.6.2(Fig. 10). For these 200 trials, all implemented functional connectivity metrics were computed. The trials were cropped to include data from 10 to 150 ms relative to the trigger. This ensured that the stimulation artifact was cut out. No ROIs were specified, meaning all-to-all networks were estimated. The clustered source space included 265 sources, which were specified as the nodes of the network. Thus, a total of 65,536 edges were calculated for each of the 200 trials and then averaged together to form the final network. The Connectivity library’s storage mode was turned on, meaning subsequent metric computations used already calculated and stored intermediate data. The sampling rate of the input data was 1,792 Hz. Frequency bins 18 to 30, which corresponded to 18 to 30 Hz (*β*-band), were averaged to calculate the functional connectivity networks. All networks were normalized and the edges representing the strongest 5% of connections were plotted. The results for the four exemplary metrics are shown for 50 and 200 trials inFigure 11. It can be observed that the metrics based on the imaginary part of the CSD, that is, IMAGCOHY and PLI, were able to detect the expected functional connection between the two activation areas S1 and S2 as strongest connections, however, only for a large number of trials. Phase- and amplitude-based metrics, such as COH, were not able to achieve the same results. Their strongest connections were already fixed to some very short-range spurious connections after a smaller number of trials and remained the same even as more trials were added. The same is true for the time domain-based metric XCOR. Figure SM 4 in theSupplementary Materialshows that this observed trend also holds true for the other metrics.

**Fig. 11.**
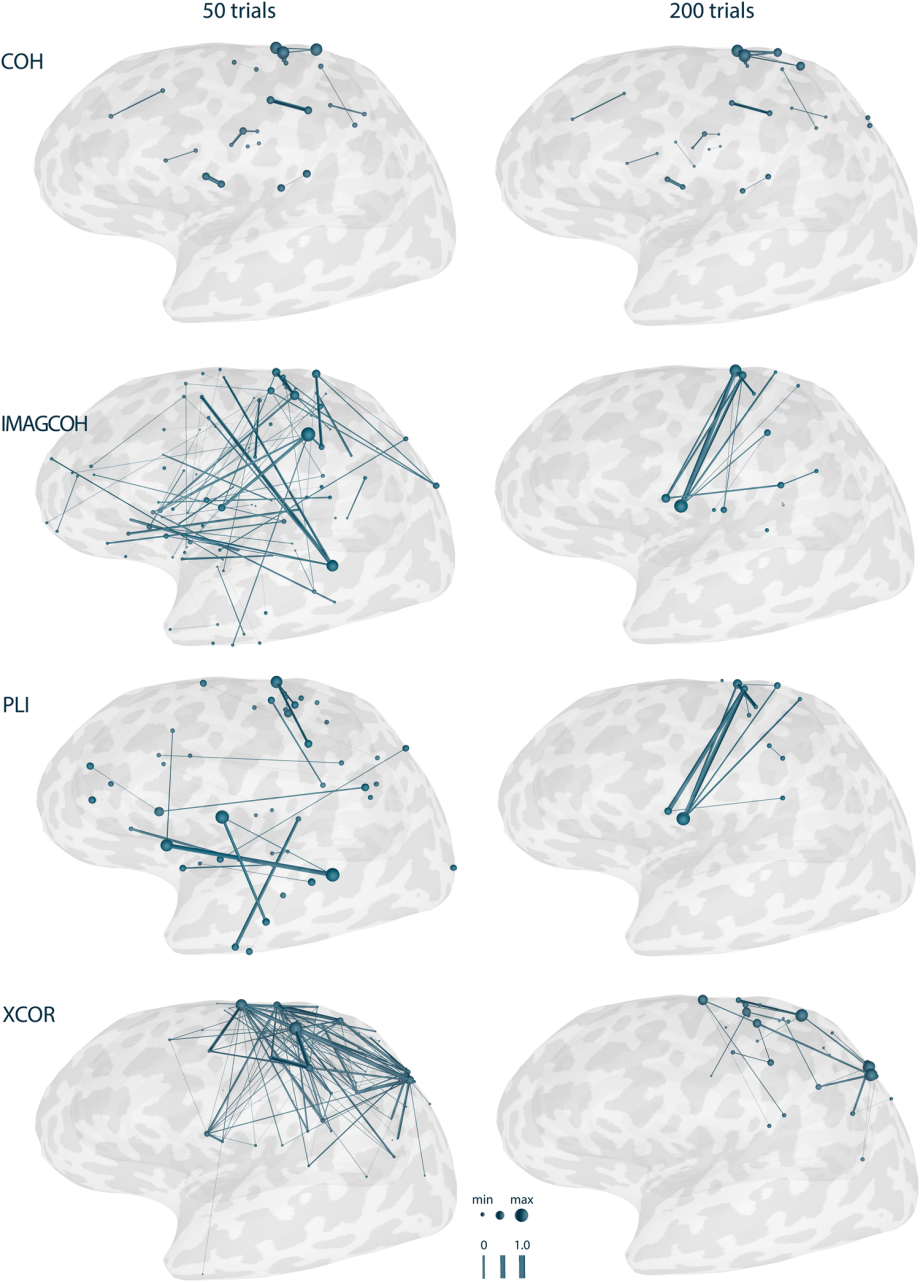
Results for functional connectivity metrics implemented in the new Connectivity library based on right-hand median nerve stimulation. Results for 50 and 200 trials are presented. Only the edges representing the strongest 5% of connections are plotted. Network nodes are plotted as spheres and edges are represented as tubes connecting the nodes. Edge strength and node degree are represented by their diameter. Note that the nodes’ sphere diameters are normalized by the node with the maximal value in the thresholded network. Results for all metrics can be found in Figure SM 4 in theSupplementary Material.

**Fig. 12.**
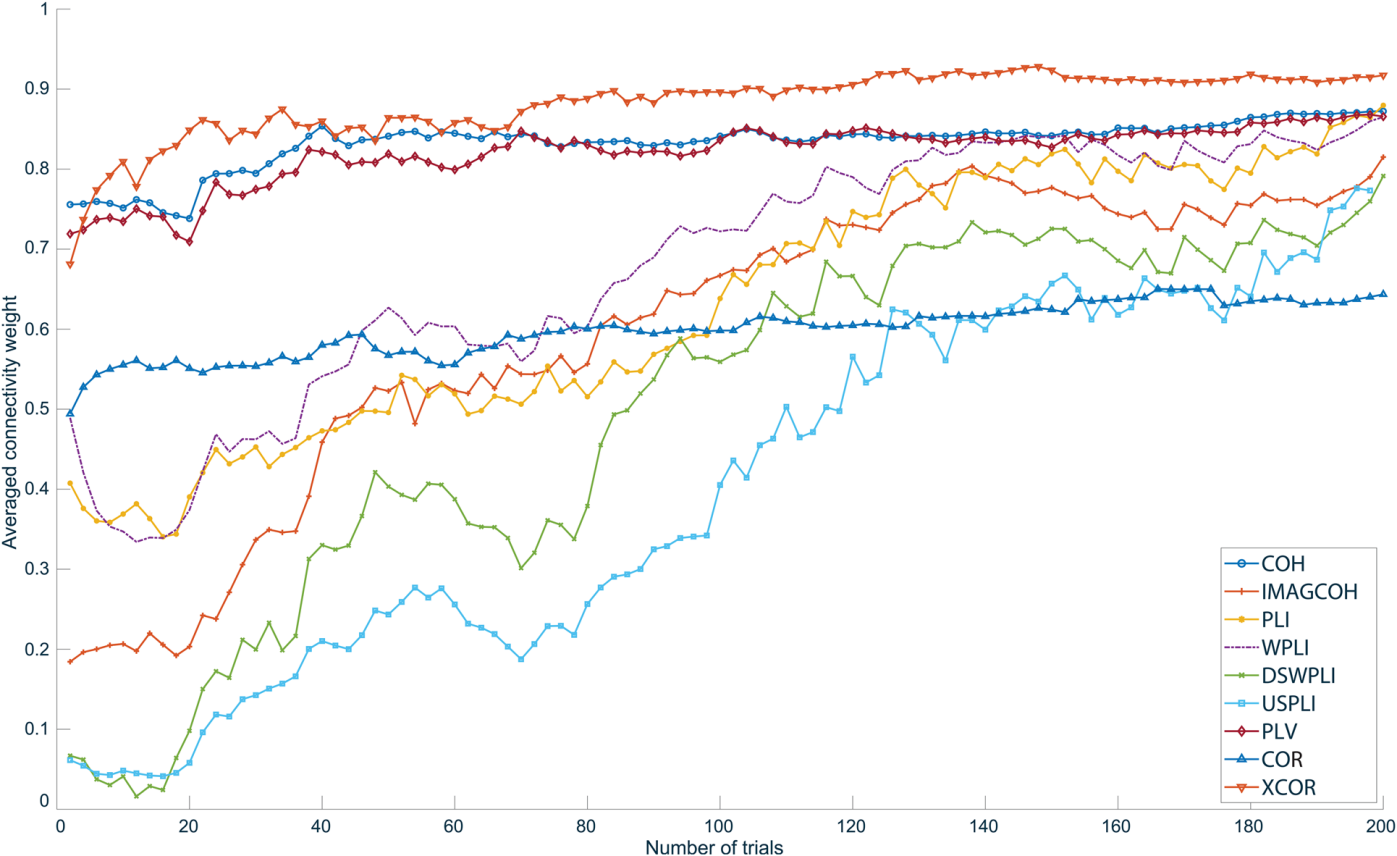
The relationship between the final networks at 200 trials and networks calculated at different number of trials. The networks were estimated for all metrics and right-hand median nerve stimulation in an offline scenario.

Figure 12presents the convergence of each metric with respect to the number of trials. As described before, for each metric, the 20 strongest weighted edges at 200 trials were identified. The same edges and their averaged weights were computed for different number of trials subsequently. Metrics based on the imaginary part of the CSD stabilized much slower than XCOR, COH, and PLV. The COR metric resulted in an almost stable average weight over all investigated number of trials. Again, note that the convergence with larger number of trials does not reflect the correctness of the results; it merely reflects how quickly the detected stable network is established.

#### 3.2.3 Online functional connectivity evaluation

**Fig. 13.**
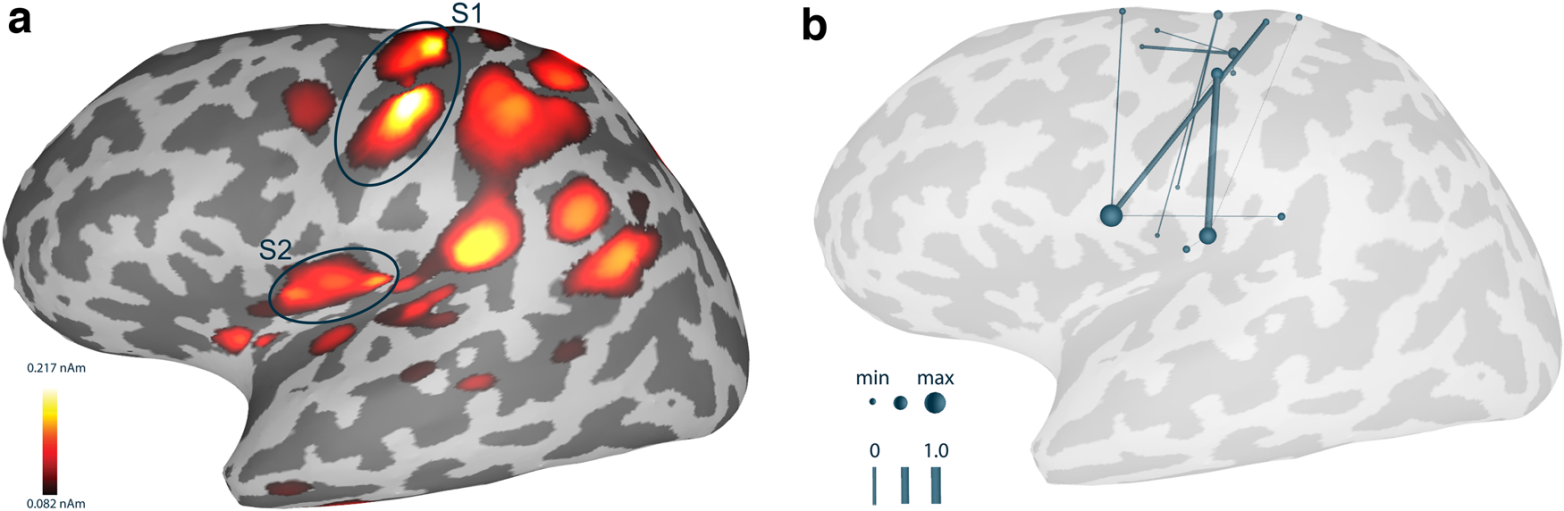
(a) One trial plotted as a 2D layout for median nerve stimulation. The result was produced in real time, and the single trial RTC-MNE result for time point 35 ms is depicted. The cortical-constrained interpolation method was used to plot the activity. The S1 and S2 areas are indicated by blue circles. The threshold was chosen to show only the strongest 75% of activities. (b) Visualization of an IMAGCOHY network estimated based on 200 good trials processed by the online connectivity pipeline. Only the edges representing the strongest 25% of connections are plotted. Network nodes are plotted as spheres and edges are represented as tubes connecting the nodes. Edge strength and node degree are represented by their diameter. Note that the nodes’ sphere diameters are normalized by the node with the maximal value in the thresholded network.

The source activity for the 265 sources was estimated for every incoming trial.Figure 13shows the RTC-MNE result for a single trial. Although plotted for a single trial, an activation in both S1 and S2 was detected. Note that the absolute value of the source activation was plotted.

Based on the RTC-MNE result, the input data stream and its including data matrices had a dimension of 265 × 250. The functional connectivity for an all-to-all network consisting of 265 nodes was calculated. The intermediate data for each trial was stored, which meant the costly computations were done only once per trial. Frequency bins 0 to 50 were calculated and bins 18 to 30, corresponding to 18 to 30 Hz, were averaged to compute the edge weights. Each newly computed trial was added to the overall network. The visualization was updated every time a new network had been calculated. The different functional connectivity metrics were switched on the fly during the measurement session. The network was normalized based on the maximum edge weight. For visualization, the threshold was set to include the strongest 10% of edges only (Fig. 13). A functional connection between the two regions of interest, S1 and S2, could be observed in real time for the frequency band 18 to 30 Hz.

The performance of the presented online functional connectivity pipeline implemented in MNE Scan depends on several parameters including the sampling frequency, block size, number of measurement channels, and the processing as well as visualization complexity of the chosen pipeline. To demonstrate these dependencies, the performance of the pipeline is presented inFigure 14. The data were recorded at a sampling frequency of 1,792 Hz. Two scenarios with the same data set streamed with different block sizes (500 sp and 1,000 sp) were examined, resulting in different performance requirements for the overall pipeline timings. For example, choosing a block size of 500 sp at a 1,792 Hz sampling frequency results in a maximum allowed processing time of 280 ms. Not meeting this time requirement would introduce an overall delay and drag down the processing pipeline. The testing specifications were the same as inEsch et al. (2018), except for the higher sampling frequency.

**Fig. 14.**
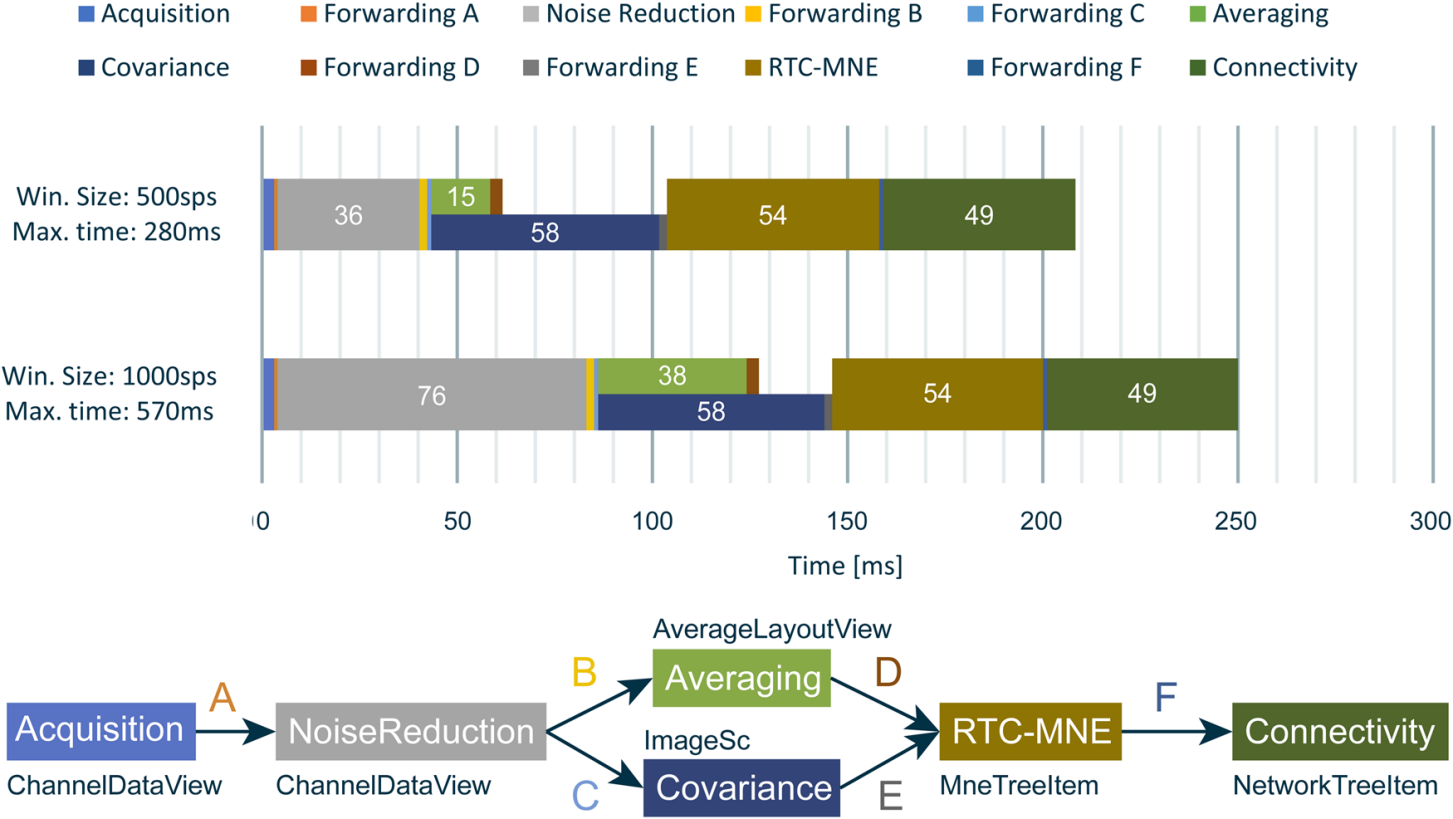
The timing values in ms for each processing step correspond to different block sizes and maximum allowed time limits. The lower plot indicates the different circular buffers between the plug-ins and the name of the corresponding visualization routine.

The timing values inFigure 14show that for both window sizes and maximum allowed processing times, MNE Scan is indeed able to execute all processing steps online. This means the overall data pipeline is not slowed down by one of the individual processing steps. Note that the averaging and covariance processing tasks are performed in parallel and not subsequent to each other. Their results are then forwarded independently to the RTC-MNE plug-in.

## 4 Discussion

The goal of this study was to present the efficient implementation of EEG/MEG functional connectivity metrics that support the computation of all-to-all connectivity networks in an easy-to-use toolbox for online EEG/MEG analysis, and demonstrate the applicability in realistic scenarios. Many previous studies restricted the number of nodes to a few ROIs in order to reduce the computational complexity or to suppress spurious connectivity. In the presented pipeline, the problem of computational burden is addressed by introducing the efficient calculation of functional connectivity metrics. Restricting the number of nodes requires prior knowledge about the process to be investigated and might result in missing out on important network nodes outside the selected ROIs. Therefore, it is desirable to include as many nodes as possible on the one hand, while keeping a meaningful and stable network on the other hand, which remains a challenge with current functional connectivity metrics. The median nerve stimulation inflicted in the proof-of-principle measurement produced a strong enough response and functional connectivity network to be estimated in the relatively large assumed all-to-all network.

In this work, all spectral metrics were implemented based on the CSD and PSD. PLV, PLI, USPLI, WPLI, and DSWPLI can be calculated based only on the CSD of the analytic signal depending either only on the phase of the signal (PLV, PLI, USPLI) or additionally weighting with the magnitude of the imaginary part of the CSD (WPLI, DSWPLI) (Vinck et al., 2011). IMAGCOHY and COH are most efficiently computed involving both CSD and PSD. From a performance standpoint and the fact that the metrics should be able to use intermediate data produced by other metrics, they should ideally share a large portion of their computational routines. Basing all spectral connectivity measures on CSD and PSD achieved that goal. This ensures an efficient and online capable implementation as well as convenient switching between metrics during an ongoing measurement session.

To date, only a few studies aimed to provide implementations of functional connectivity metrics that allow for an application in online scenarios.García-Prieto et al. (2017)describe tools to compute phase synchronization measures such as IMAGCOHY, PLV, PLI, and WPLI in real time. Compared with the present work, the authors chose a slightly different approach by first filtering the signal to the frequency band of choice and then generating the analytic signal via the Hilbert Transform. This enables the investigation of time-resolved functional connectivity by analyzing the instantaneous phase and amplitude. In consequence, the subsequent functional connectivity estimation relies on the ability to design very narrow-band temporal filters. Designing such filters is a nontrivial task and applying them involves another costly FFT convolution. Additionally, most of the analysis must be repeated if a different frequency band is selected. In an online processing pipeline doing trial-resolved connectivity estimation, a change of the frequency band would mean that all trials would need to be processed again.

Hwang et al. (2007)andHwang et al. (2011)implemented a functional connectivity EEG pipeline based on source-level data, and the source estimation was realized with MNE. The functional connectivity metric was chosen to be a simple COR and included 12 nodes.Billinger et al. (2013,^2015^) describe an Independent Component Analysis (ICA)-based pipeline that can estimate effective connectivity on a small number of nodes.Mullen et al. (2013)andMullen et al. (2015)focus on a more advanced online pipeline including distributed source estimation, effective connectivity with a small number of nodes, and an online capable visualization including a GUI.

In comparison, the pipeline proposed in this work includes a device-independent acquisition with modular processing steps. These processing steps include temporal filtering, averaging, covariance estimation, source estimation, and connectivity analysis of large functional networks (>200 nodes). Additionally, the result of each step can be visualized in 2D or 3D. To the best of our knowledge, the proposed pipeline is the first to combine device independence, online preprocessing, online distributed source-level reconstruction, online estimation and visualization of large functional connectivity networks, and GUI usage.

Except forGarcía-Prieto et al. (2017), no detailed performance values for online capable functional connectivity metrics were presented in prior publications.García-Prieto et al. (2017)evaluated the performance of the metrics based on a single trial with random data for different sample sizes and number of nodes. The hardware used to generate the performance values is different from the system in this work that provided 24 logical cores compared with 40 inGarcía-Prieto et al. (2017). The performance of the Connectivity library presented in this work outperforms the implementation presented inGarcía-Prieto et al. (2017), even though the computations were performed on a less powerful setup. However, this does not hold for window sizes smaller than 800 sp. This could be due to the extra code necessary to set up the data structures for multithreading in the implementation presented here. Note that the multithreading setup is also executed when only one or a number of trials smaller than the number of CPU cores are processed.

The storage mode of the new Connectivity library enables the reuse of intermediate data generated in previous computations. This means that for every newly added trial, only the evaluation of this single trial is necessary to update the connectivity network. However, it is important to mention that some code sections need to be run in order to check whether intermediate data from existing trials are available. This overhead needs to be considered when measuring the performance with the storage mode activated. With a large number of trials and analyzing multiple different metrics, the memory load corresponding to the stored intermediate data can be high. For this reason, the storage mode should only be used in demanding online setups together with hardware including a high memory capacity. In offline cases, the storage mode should be turned off in order to save memory. This will make the switching between metrics and recalculating metrics slower in favor of lower memory consumption.

The simulation-based source estimation results, both for the full and clustered source space, showed correct activity patterns in the selected ROIs. In the case of Label 2, the source activity spreads to the temporal lobe, seeFigure 7, which can be explained by the curved cortical mantle. It is important to remember that the activity was visualized on an inflated surface. Whereas the visualization on the inflated surface gives the impression of a significant distance between the simulated source L2 and the found activation area on the temporal lobe, this distance is not present in the actual source space/cortex surface. The strongest active sources in both ROIs revealed the appropriate phase-lagged time courses (Fig. 7).

The source reconstructed signals were used to validate the newly implemented metrics. All-to-all networks were computed between 243 nodes. The results show that the implemented metrics correctly identify functional connectivity between the two specified labels in the simulated data. Based on the fact that a 90° phase shift between the 2 labels’ signal courses was introduced, it was to be expected that metrics based on the imaginary part of the CSD would be better suited to detect the signals’ similarity. As shown byNolte et al. (2004), zero phase lag connectivity is discarded by IMAGCOHY and other metrics using the imaginary part of the CSD. This makes them more robust against the effects of volume conduction. This behavior is clearly reflected in the results presented inFigure 8for the IMAGCOHY, PLI, USPLI, and WPLI. Although XCOR is not a spectral metric, it does account for temporal structure. That is why XCOR was able to detect the connectivity of the phase-lagged signals as well. All other spectral metrics and COR were not able to identify the true connectivity as one of the strongest connections, but these were dominated by very short-range spurious connections. However, it might be possible to reconstruct the true connectivity with these metrics when excluding such connections from the analysis. These results confirm similar findings and previous discussions regarding the pitfalls of functional connectivity metrics (Bastos & Schoffelen, 2016;Palva et al., 2018).

Figure 9shows that all spectral metrics and XCOR converge relatively quickly toward their final network structure. However, it can be seen that COH and PLV converge slightly faster than the other metrics. Note that in this simulated case, no additional sources were active except for the simulated ones. Consequently, this scenario is an idealized one with only the added noise interfering with the true connectivity.

The results of the proof-of-principle evaluation using realistic data show that meaningful functional connectivity networks can be reconstructed if a sufficient number of trials is considered.Figures 11and13, and Figure SM 4 in theSupplementary Materialshow that for 200 trials, all metrics that suppress instantaneous connectivity (IMAGCOH, PLI, USPLI, WPLI, DSWPLI) identify the strongest connectivity in the*β*-band (18 to 30 Hz) between contralateral S1 and S2, as it is expected from the literature (Hari & Forss, 1999;Simoes et al., 2003). It is remarkable that none of the metrics identified this connection at 50 trials. For the other metrics, the strongest connections are dominated by very short-range connectivity due to field spread covering the true connectivity.Figure 12underlines that all metrics based on the imaginary part of the CSD stabilized only slowly and required more than 100 trials to converge for the realistic data. However, exactly for these metrics, the strongest connections corresponded to the true connectivity.

Besides the connectivity between contralateral S1 and S2,Simoes et al. (2003)also detected phase locking between the contralateral S1 and the ipsilateral S2. Even though our analysis included both hemispheres, no such connectivity involving the ipsilateral hemisphere could be identified, which could have several reasons.Simoes et al. (2003)performed their analysis for planar gradiometers, which have a focal sensitivity profile and prevent cross-talk. Prior to their connectivity analysis,Simoes et al. (2003)identified the sensors with the strongest response in the S1 and S2 regions to obtain optimal results. Nevertheless, the connectivity between contralateral S1 and ipsilateral S2 was clearly weaker than that between contralateral S1 and S2. In contrast, our source space analysis relied on a clustered source space with a relatively low resolution. In consequence, activity in both S1 and S2 was most probably not represented by a single perfectly matching source but by multiple extended sources (seeFig. 10) that are also influenced by cross-talk from other sources. Thus, strength of the reconstructed sources—and in result also the SNR and the estimated functional connectivity—is further reduced for each individual source. Obviously, this diminishes the chances to correctly identify this connectivity.

A rather slow convergence of connectivity metrics based on CSD and PSD is predicted by theoretical results, which have shown that the confidence intervals for coherence and phase only reduce with the inverse square root of the number of disjunct time intervals, which corresponds to the number of trials in our analysis (Halliday et al., 1995;Rosenberg et al., 1989;Wang & Tang, 2004). The length of each trial does not influence this convergence but only increases the frequency resolution. As a consequence of this rather slow convergence, especially also the suppression of spurious connectivity due to instantaneous connectivity for metrics such IMAGCOHY, PLI, or WPLI is only effective for a higher number of trials. For a smaller number of trials, random fluctuations might still contribute to the imaginary part, which are also expected to vanish with the inverse square root of the number of trials (Nolte et al., 2004;Nolte & Marzetti, 2014). This is exemplarily demonstrated by the clearly worse convergence of IMAGCOHY compared with COH. Also the involvement of the absolute value in PLI and WPLI might further affect the convergence. In conclusion, the relatively high number of trials required for some of the connectivity metrics, especially also those suppressing instantaneous connectivity, might not be achievable in practice for some applications. In such situations, it might be preferable to apply metrics that converge faster, such as XCOR, and prevent spurious connectivity, for example, by excluding very short-range connectivity or defining ROIs based on prior knowledge.

The online results are comparable with those created offline prior to the online session (Fig. 11). This is an expected result since the offline and online results are computed using the exact same implementations with the only difference being that the latter was embedded in an online scenario with stringent time constraints.Figure 12shows that metrics projected onto the imaginary plane take much longer to stabilize their final structure, but show meaningful connectivity between S1 and S2. Metrics based on both the real and imaginary part of the CSD need fewer trials to reach their final network structure but are prone to volume conduction effects and do not show significant connectivity between S1 and S2. The difference in this final network generation speed can be explained by the fact that IMAGCOHY, PLI, USPLI, DSWPLI, and WPLI effectively discard information when being projected onto the imaginary plane. This is why they are more prone to noise and need more trials to produce clear results. On the contrary, metrics such as COH and PLV converge faster toward their final structure since they are not reduced in dimensionality. In contrast to the simulation study, COR and XCOR do not produce any plausible results when evaluating actual measurement data.

The overall timings of the final pipeline meet the online requirements as shown inFigure 14. The presented timing values are based on operating systems not able to guarantee hard real-time requirements: Windows, macOS, and Linux hide specific thread prioritization and handling from the user. In order to meet stringent real-time requirements, meaning one can be certain that routines always take a predictable amount of time to run, Real-Time Operating Systems (RTOSs) can be employed. Examples of RTOSs are QNX (http://blackberry.qnx.com/en/products/neutrino-rtos/index) and RTX (https://www.intervalzero.com/). Since MNE-CPP and MNE Scan are solely based on Qt and Eigen, deploying them on an RTOS is feasible.

As reviewed and investigated in several prior publications, functional connectivity estimation is highly sensitive to a wide range of external factors and variations in preprocessing (Barzegaran & Knyazeva, 2017;Bastos & Schoffelen, 2016;Cho et al., 2015;Haufe & Ewald, 2019;Palva et al., 2018;Sakkalis, 2011;Schoffelen & Gross, 2009;Sitaram et al., 2017). These include a low SNR, coregistration, artifacts, temporal filtering, the device-to-head transformation matrix, type of baseline correction, choice of source estimation method, and head model accuracy to name the most important ones. In the proposed pipeline, trial rather than time-resolved functional connectivity was computed in order to cope with the low SNR. Moreover, SSP and temporal filtering were employed to improve the SNR. A rather simple EOG-based artifact rejection was implemented, which excluded trials if a threshold was exceeded. It was already shown in a prior work that the RTC-MNE method is capable of coping with low SNR and eases computational complexity (Dinh et al., 2015). For this reason, the RTC-MNE method was chosen as the basis for the source-space functional connectivity evaluation.

The number of necessary trials to obtain stable functional connectivity networks observed in this study may seem very (or too) high for an online application. However, multiple studies proposed algorithms for brain-state classification based on functional connectivity estimates based on few or even single trials, for example, for use in BCIs, though none of them actually applied their algorithm in an online scenario (Antonacci et al., 2019;Billinger et al., 2013;Feng et al., 2020;Rathee et al., 2017;Shamsi et al., 2021).Feng et al. (2020)demonstrated that sensor space functional connectivity estimates evaluated on different frequency bands with standard EEG preprocessing (re-referencing, temporal filtering) generate proper features for motor imagery (MI) classification, even outperforming a common spatial pattern (CSP)-based approach. In a similar scenario and with standard EEG preprocessing,Rathee et al. (2017)additionally applied source reconstruction to avoid spurious connectivity due to volume conduction, and again showed that features derived from functional connectivity estimates can improve the classification accuracy in MI tasks in comparison with established methods such as CSP. Such approaches could be implemented in MNE Scan after the implementation of the respective classifiers, which is currently a work in progress.

In future releases, besides the addition of additional features, such as the already mentioned classifiers, also the implementation of alternative methods for already implemented pipeline segments such as preprocessing, source analysis, functional connectivity estimation, and postprocessing should be considered. For source analysis, especially the implementation of beamformer approaches is of interest, as these are a frequent choice for source-space functional connectivity estimation (Brookes et al., 2011;Fuchs, 2007) andMahjoory et al. (2017)have shown the importance of using various source analysis methods to validate connectivity estimates. For functional connectivity estimation, besides COR and XCOR, the focus of the current implementation has been on phase-based measures. Popular metrics that could complement the current selection of functional connectivity measures are, for example, amplitude envelope correlation (AEC) or mutual information, or even effective connectivity metrics such as Granger causality or partial directed coherence (O’Neill et al., 2018;Schoffelen & Gross, 2019). For the analysis of dynamic functional connectivity, also the implementation of ICA, principal component analysis (PCA), or hidden Markov model (HMM) algorithms is of interest to detect underlying transient connectivity patterns (O’Neill et al., 2018). For the postprocessing, a statistical analysis or quality scores about the analyzed signals and the resulting connectivity networks could be provided (Brookes et al., 2012;Hipp et al., 2012). Of course, these considerations are subject to the possibility of a real-time capable implementation.

As an alternative approach to prevent source leakages and resulting spurious connectivity,Brookes et al. (2012),Colclough et al. (2015), andHipp et al. (2012)proposed to orthogonalize the source time courses prior to the connectivity estimation and demonstrate the benefit of this approach for the estimation of functional connectivity using AEC. However, inBrookes et al. (2012),Colclough et al. (2015), andHipp et al. (2012), the orthogonalization is performed offline for source time courses of 300, 500, and 600 s, respectively. An application for real-time functional connectivity estimation would require an update of the orthogonalization for each change of the source time courses, that is, with each added measurement block (for resting-state activity) or trial (for evoked activity). Especially for the relatively long time courses obtained in resting-state measurements, this could result in a significant computational effort, which needs to be taken into account when considering an implementation of this approach for real-time functional connectivity estimation.

## 5 Conclusion

The goal of this work was to integrate functional EEG/MEG connectivity analysis of large all-to-all networks into an online setting with a complete online pipeline including a GUI, which—to the best of our knowledge—has not been accomplished so far. A proof-of-principle measurement showed that the implemented tools are indeed able to provide online functional connectivity analysis if necessary requirements are met.

The scope of this work did not include the development of new online capable metrics or necessary hardware. However, the results of this work confirm that new connectivity metrics and/or measurement hardware might be necessary in order to move toward true real-time connectivity estimation either on a single-trial basis or to obtain an instantaneous result per sample. Nevertheless, the present work can be understood as a major step toward true real-time connectivity analysis as it demonstrated that efficient implementations of connectivity metrics enable the online processing of EEG/MEG data with existing and widely available computational resources.

The tools presented in this study allow to improve and ease access to online data monitoring for assessing data quality during rather than after the measurement making it possible to rule out experimental problems early on. Moreover, they provide the foundation for the design of novel BCI applications and neurofeedback experiments based on functional connectivity networks. Integrating connectivity estimation into a neurofeedback setup is nontrivial and often requires single-trial or instantaneous rather than averaged results. Although this work primarily focused on the online estimation of evoked responses, spontaneous data can, from a technical point of view, be analyzed with the implemented tools, but estimating functional connectivity for spontaneous or resting-state data and related feature classification is the subject of ongoing research efforts (Billinger et al., 2013;Colclough et al., 2016;Feng et al., 2020;Garcés et al., 2016;Rathee et al., 2017;Shamsi et al., 2021). As laid out in theSection 4, for a reliable application of such pipelines based on MNE Scan in practice, further implementations and evaluations are necessary.

## Supplementary Material

Supplementary Material

Supplementary Material

## Data Availability

The simulated data used inSection 2.6.1can be found in theSupplementary Materialtogether with a Python script to recreate the data. The MIND data set*sub-mind010*used inSection 2.6.2can be downloaded from the OpenNEURO database athttps://openneuro.org/datasets/ds004107/versions/1.0.0, which can also be found under the permanent digital object identifier (DOI)https://doi.org/10.18112/openneuro.ds004107.v1.0.0. Additional information can be found athttps://mne.tools/mne-bids-pipeline/1.4/examples/ds004107.html. MNE Scan is part of the MNE-CPP project. Binaries can be downloaded athttps://mne-cpp.github.io/pages/download/download.html, and the source code repository is available athttps://github.com/mne-tools/mne-cpp. The MNE-CPP repository contains minimal working examples in the*examples*-subfolder, which can be easily compiled by selecting the option “BUILD_EXAMPLES” in the MNE-CPP cmake. To recreate the connectivity visualizations and performance evaluations, the examples*ex_connectivity*and*ex_connectivity_performance*can be used. Furthermore, the example*ex_connectivity_comparison*allows for a direct comparison of the results for the different connectivity measures. For this manuscript, the examples were last tested using commit*0d92e66*and compiled using Qt 5.15. To reproduce the results from the manuscript (obey that there is a certain variation due to the nondeterministic behavior of the clustering algorithm), use the call **%binary***--fileIn=*%pathToFile*/sample_twosource-meg-simulated-raw.fif* *--eve=*%pathToFile*/sample_twosource-meg-simulated-eve.fif* *--cov=*%pathToFile*/sample_twosource-meg-simulated-cov.fif* *--sourceLocMethod=MNE --tmin=-0.05 --tmax=0.16 --snr=3.27 --aveIdx=1* *(--freqmin=0 --freqmax=50)* Minimum and maximum frequencies have to be indicated for*ex_connectivity_comparison*only. Additionally, the*MNE-sample-data*have to be placed in the MNE-CPP directory, a description how to download them and where to place them is contained in the GitHub-repository.
