# Supplementary material for: Online functional connectivity analysis of large all-to-all networks in MNE Scan
Source: Imaging Neurosci (Camb). 2024 Sep 25;2:imag-2-00296. doi: 10.1162/imag_a_00296 (PMC12290595; doi:10.1162/imag_a_00296)
Supplement: Supplementary Material [file imag_a_00296-supp.pdf]

Supplementary Material 1 Functional Connectivity Metrics

Supplementary Material 1.1 Time Domain

For two signals in time domain, $x(t)$ and $y(t)$, we define

Correlation (COR)

$$COR_{xy} = \frac{\sum_{t=1}^{N}(x(t)) - \bar{x})(y(t) - \bar{y})}{\sqrt{\sum_{t=1}^{N}(x(t) - \bar{x})^2 \sum_{t=1}^{N}(y(t) - \bar{y})^2}} \tag{1}$$

Cross Correlation (XCOR)

$$XCOR_{xy}(\tau) = \frac{\sum_{t=1}^{N-\tau}(x(t+\tau)) - \bar{x})(y(t) - \bar{y})}{\sqrt{\sum_{t=1}^{N-\tau}(x(t+\tau) - \bar{x})^2 \sum_{t=1}^{N}(y(t) - \bar{y})^2}} \tag{2}$$

$$\{XCOR_{xy} \in \mathbb{R} | -1 \leq COR_{xy} \leq 1\} \tag{3}$$

Supplementary Material 1.2 Frequency Domain

For two signals in the frequency domain, $x(\omega)$ and $y(\omega)$, with signal magnitudes $A_x(\omega)$ and $A_y(\omega)$ and phases $\Phi_x(\omega)$ and $\Phi_y(\omega)$, we define the power spectral density (PSD) and cross spectral density (CSD)

$$PSD_x(\omega) = A_x^2(\omega) \tag{4}$$

$$CSD_{xy}(\omega) = x(\omega)y^*(\omega) \tag{5}$$

$$= A_x(\omega)A_y(\omega)e^{i(\Phi_x(\omega)-\Phi_y(\omega))} \tag{6}$$

For K trials, we define the following connectivity metrics:

Coherency (COHY)

$$COHY_{xy}(\omega) = \frac{\frac{1}{K}\sum_{k=1}^{K} A_x(\omega,k)A_y(\omega,k)e^{i(\Phi_x(\omega,k)-\Phi_y(\omega,k))}}{\sqrt{\left(\frac{1}{K}\sum_{k=1}^{K} A_x^2(\omega,k)\right)\left(\frac{1}{K}\sum_{k=1}^{K} A_y^2(\omega,k)\right)}} \tag{7}$$

$$= \frac{\frac{1}{K}\sum_{k=1}^{K} CSD_{xy}(\omega,k)}{\sqrt{\left(\frac{1}{K}\sum_{k=1}^{K} PSD_y(\omega,k)\right)\left(\frac{1}{K}\sum_{k=1}^{K} PSD_y(\omega,k)\right)}} \tag{8}$$

Coherence (COH)

$$COH_{xy}(\omega) = |COHY_{xy}(\omega)| \tag{9}$$

Imaginary Part of Coherency (IMAGCOHY)

$$IMAGCOHY_{xy}(\omega) = Im(COHY_{xy}(\omega)) \tag{10}$$

Phase Locking Value (PLV)

$$PLV_{xy}(\omega) = \left|\frac{1}{K}\sum_{k=1}^{K} e^{i(\Phi_x(\omega,k)-\Phi_y(\omega,k))}\right| \tag{11}$$

$$= \left|\frac{1}{K}\sum_{k=1}^{K} \frac{CSD_{xy}(\omega)}{|CSD_{xy}(\omega)|}\right| \tag{12}$$

Phase Lag Index (PLI)

$$PLI_{xy}(\omega) = \left|\frac{1}{K}\sum_{k=1}^{K} sign(Im(CSD_{xy}(\omega)))\right| \tag{13}$$

Unbiase Squared PLI (USPLI)

$$USPLI_{xy}(\omega) = \frac{K\,PLI_{xy}^2 - 1}{K-1} \tag{14}$$

Weighted PLI (WPLI)

$$WPLI_{xy}(\omega) = \frac{|\frac{1}{K}\sum_{k=1}^{K} Im(CSD_{xy}(\omega))|}{\frac{1}{K}\sum_{k=1}^{K} |Im(CSD_{xy}(\omega)|} \tag{15}$$

Debiased Squared WPLI (DSWPLI)

$$DSWPLI_{xy}(\omega) = WPLI_{xy}^{2}(\omega) \tag{16}$$

Supplementary Material 2 Supplementary Figures

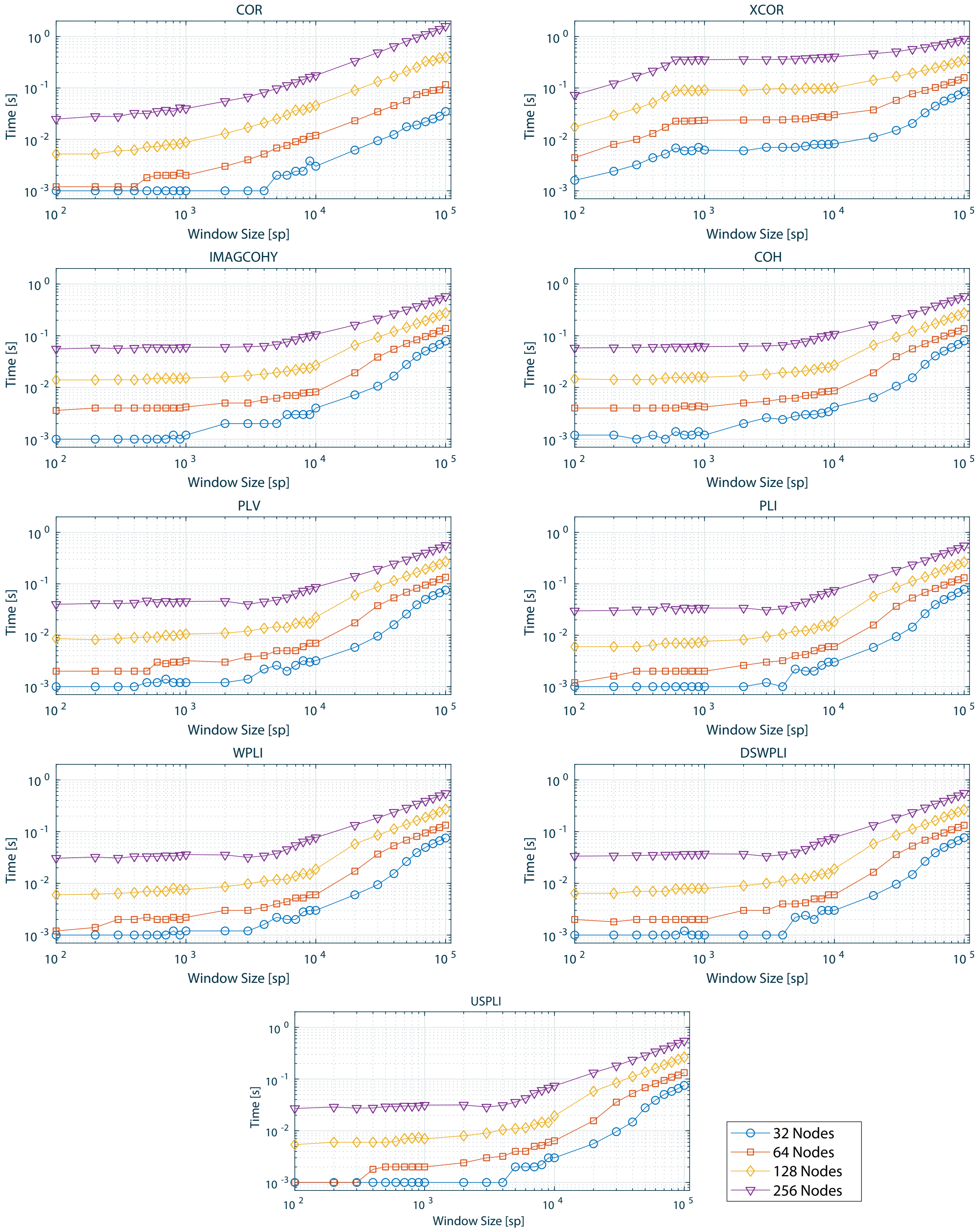

Figure SM 1: Computational timing values in seconds for one trial and different window sizes in samples (sp) as well as number of computed nodes (y-axis in logarithmic scale). Computations were generated averaged over five repetitions.

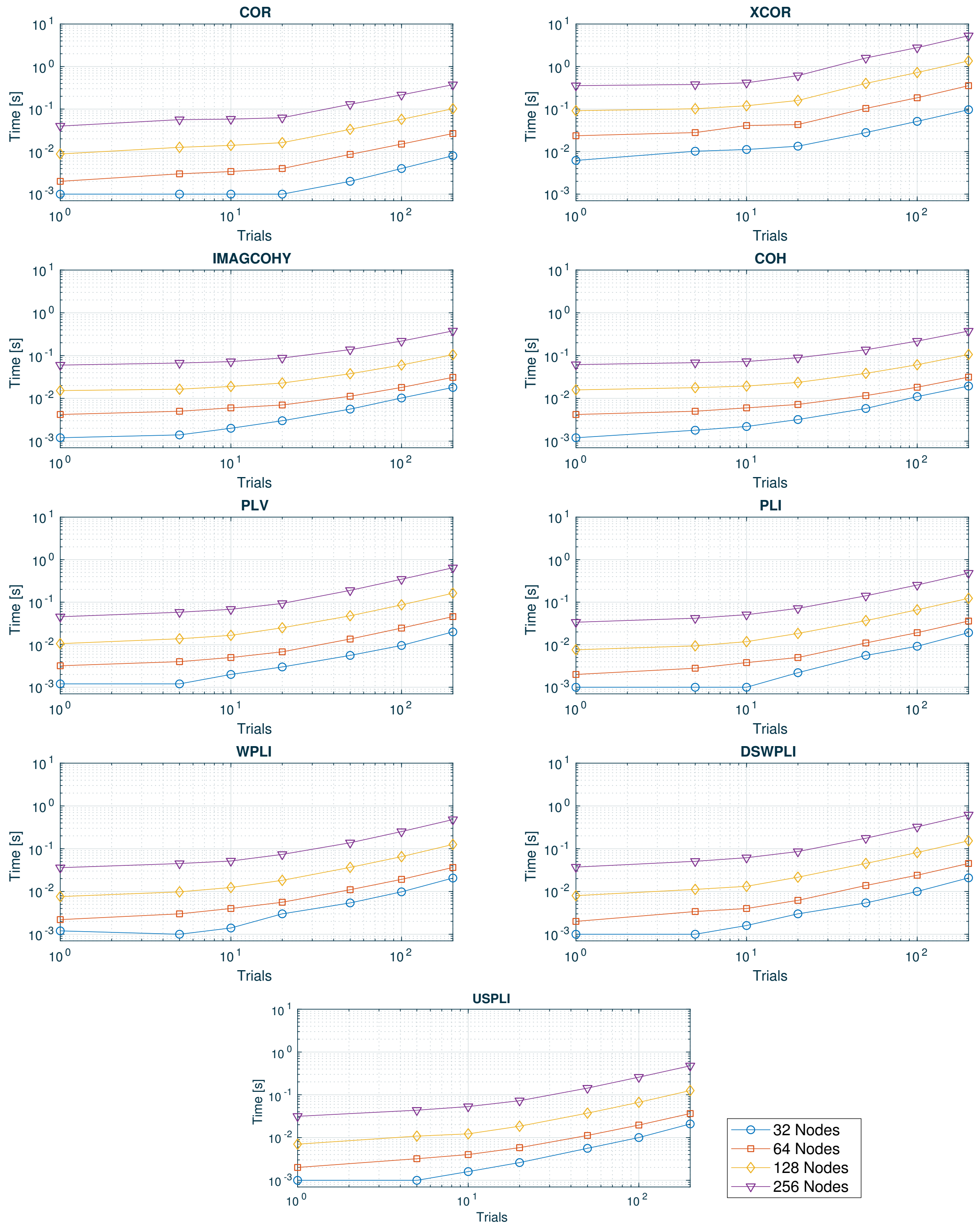

Figure SM 2: Computational timing values in seconds for 1,000 sp and different number of trials as well as computed nodes (y-axis in logarithmic scale). Computations were generated averaged over five repetitions.

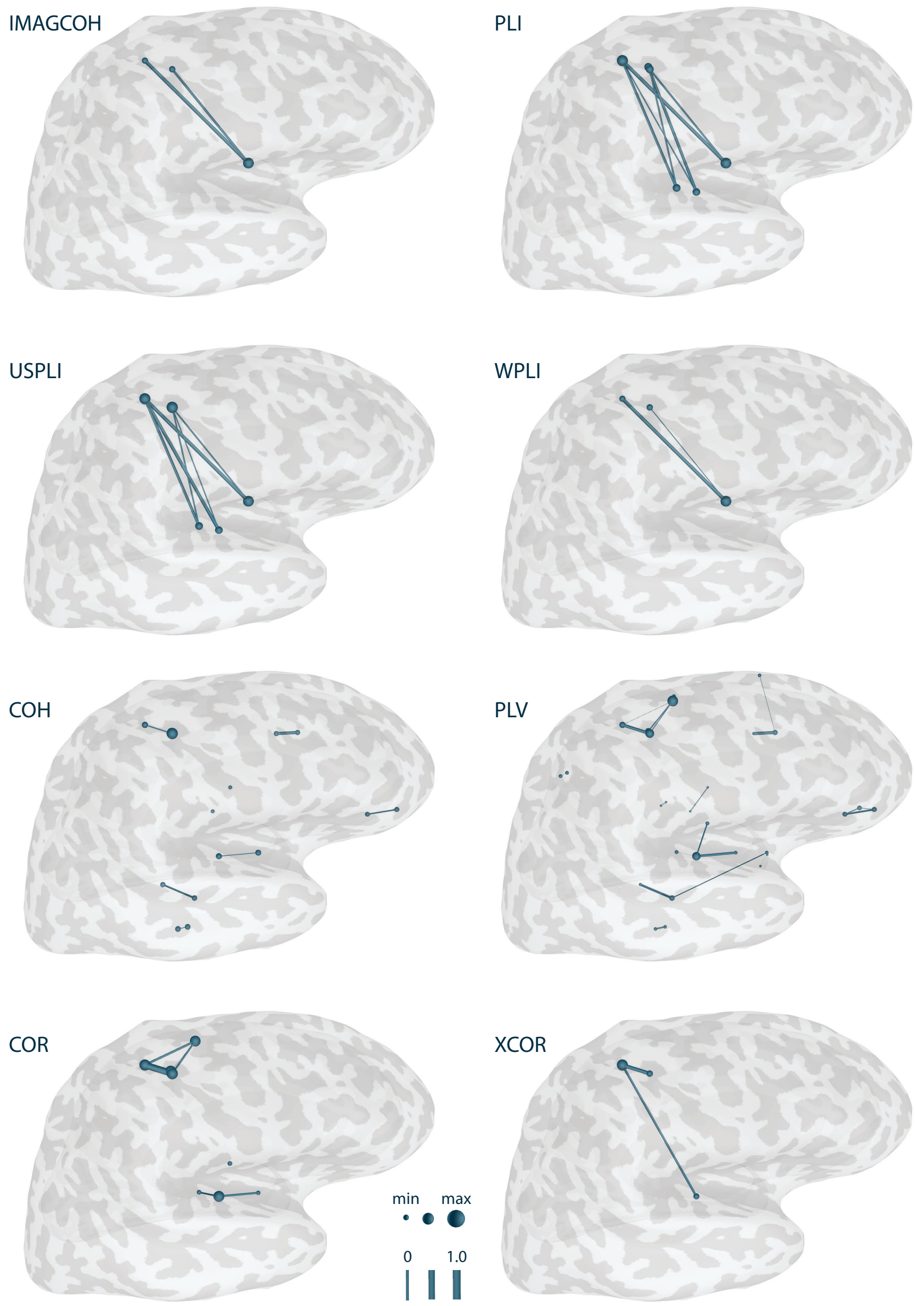

Figure SM 3: Functional connectivity networks for different metrics based on simulated data. The RTC-MNE method was used to compute the source activity. The number of trials was 200. Network nodes are plotted as spheres and edges are represented as tubes connecting the nodes. Edge strength and node degree are represented by their diameter. Please note that the nodes' sphere diameters are normalized relative to the node with the maximal value in the thresholded network.

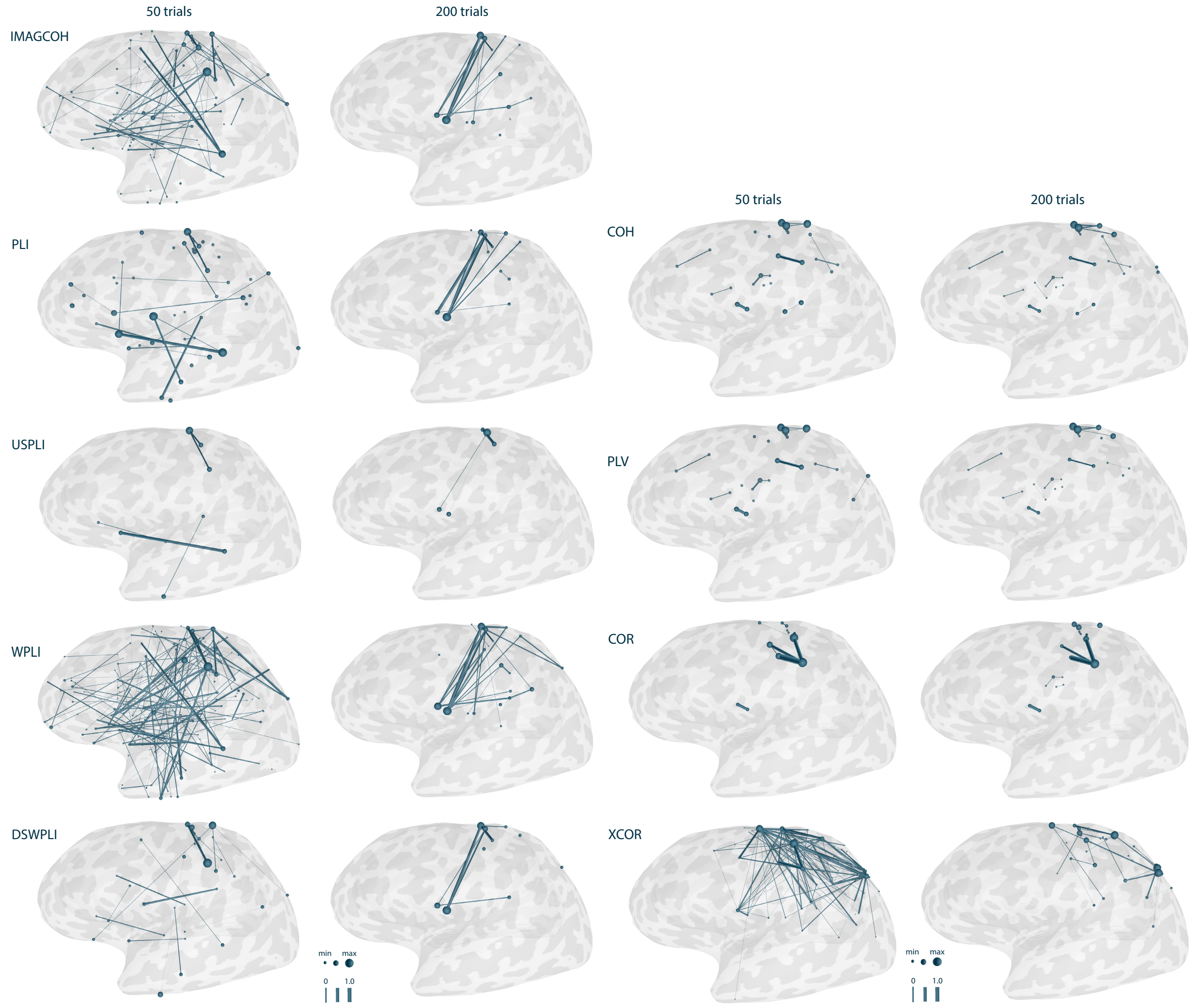

Figure SM 4: Results for functional connectivity metrics implemented in the new Connectivity library based on right-hand median nerve stimulation. Results for 50 and 200 trials are presented. Only the edges representing the strongest 5% of connections are plotted. Network nodes are plotted as spheres and edges are represented as tubes connecting the nodes. Edge strength and node degree are represented by their diameter. Please note that the nodes' sphere diameters are normalized relative to the node with the maximal value in the thresholded network.

Supplementary Material 3 Test Data

Simulated Data Set	*sample_twosource-meg-eeg-simulated-raw.fif*,
	sample_twosource-meg-eeg-simulated-eve.fif,
	sample_twosource-meg-simulated-cov.fif
Python Script	*mne-python-simulated-connectivity.py*,
	mne-python-simulated-connectivity.ipynb
